# A Method for Metagenomics of *Helicobacter pylori* from Archived Formalin-Fixed Gastric Biopsies Permitting Longitudinal Studies of Carcinogenic Risk

**DOI:** 10.1371/journal.pone.0026442

**Published:** 2011-10-21

**Authors:** Zongli Zheng, Anders F. Andersson, Weimin Ye, Olof Nyrén, Staffan Normark, Lars Engstrand

**Affiliations:** 1 Department of Medical Epidemiology and Biostatistics, Karolinska Institutet, Stockholm, Sweden; 2 Department of Microbiology, Tumor and Cell Biology, Karolinska Institutet, Stockholm, Sweden; 3 Science for Life Laboratory, KTH Royal Institute of Technology, Stockholm, Sweden; 4 Swedish Institute for Communicable Disease Control, Solna, Sweden; Technische Universität München, Germany

## Abstract

The human microbiota has come into focus in the search for component causes of chronic diseases, such as gastrointestinal cancers. Presumably long induction periods and altered local environments after disease onset call for the development of methods for characterization of microorganisms colonizing the host decades before disease onset. Sequencing of microbial genomes in old formalin-fixed and paraffin-embedded (FFPE) gastrointestinal biopsies provides a means for such studies but is still challenging. Here we report a method based on laser capture micro-dissection and modified Roche 454 high-throughput pyrosequencing to obtain metagenomic profiles of *Helicobacter pylori*. We applied this method to two 15 year old FFPE biopsies from two patients. Frozen homogenized biopsies from the same gastroscopy sessions were also available for comparison after re-culture of *H. pylori*. For both patients, *H. pylori* DNA dissected from FFPE sections had ∼96.4% identity with culture DNA from the same patients, while only ∼92.5% identity with GenBank reference genomes, and with culture DNA from the other patient. About 82% and 60% of the predicted genes in the two genomes were captured by at least a single sequencing read. Along with sequences displaying high similarity to known *H. pylori* genes, novel and highly variant *H. pylori* sequences were identified in the FFPE sections by our physical enrichment approach, which would likely not have been detected by a sequence capture approach. The study demonstrates the feasibility of longitudinal metagenomic studies of *H. pylori* using decade-preserved FFPE biopsies.

## Introduction

It is a formidable challenge – but of increasing interest – to identify microbial virulence factors that might be tentatively involved in the causation of chronic diseases such as cancer. The microbes that are detected at the time of disease diagnosis may not reflect the microbiota that was present when the disease process was initiated. If the disease or its precursor lesions change the growth conditions for a causative microbial strain, this strain may either become displaced by competing microorganisms or change its molecular makeup to adapt to the altered environment. This is particularly true for *Helicobacter pylori* (*H. pylori*)-associated gastric cancer because of two preconditions. First, *H. pylori* harbor a plastic genome with a high recombination rate. A previous study estimated that about half of the *H. pylori* genome could be exchanged during the course of 41 years [1]. Second, the latency period between the presumed carcinogenic actions of *H. pylori* infection and cancer diagnosis is believed to be decades, during which time profound changes typically occur in the microorganism's niche [2]. Hence, collections of samples obtained before or in the early stage of disease development, or more specifically samples incidentally collected for reasons unrelated to the outcome disease and with follow-up for a period of time that exceeds the expected latency, would potentially be valuable resources for identifying relevant and important virulence factors. Ideally, archived frozen biopsies would provide high quality DNA for such analyses, but such material is not collected in routine health care. Available samples, if any, are clearly too few for longitudinal epidemiological studies of rare diseases such as site-specific cancer. Such studies typically require many thousands of non-malignant samples and follow-up for decades to accumulate just a handful of incident cases. Thus, routinely collected and archived formalin-fixed and paraffin-embedded (FFPE) biopsies at pathology clinics constitute an appealing potential alternative.

However, the nature of the FFPE samples has hampered their usefulness. Obstacles in sequencing *H. pylori* metagenomes from FFPE samples include minute amounts of DNA, DNA degradation, and – most difficult – interference by human DNA. The minute amounts of highly degraded DNA limit the use of the multiple displacement amplification method, which has been demonstrated to have least amplification bias among current methods [3]. For enrichment of target sequences, commercial methods have been available and their performances have been improved steadily over the past few years. One recent comparison of the performances of three commercial methods [4] showed that the number of reads that aligned to the region of interest over total number of reads ranged from 52% to 59%. The percentage of targeted region being sequenced increases with increasing sequencing depth but levels off at certain point where little gain is achieved compared to the amount of additional data. Similarly for all the three methods compared, only about 1.75 Mb out of a 2.61-Mb target region (the level off point, corresponding to a proportion of ∼67%,) was sequenced after sequencing 100 Mb to 300 Mb [4] of enriched DNA. Further, these enrichment strategies rely on current knowledge in target genomes to enable design and synthesis of probes for capturing target genomic sequences in liquid- or solid-phase. This approach might not give optimal performance in the enrichment of genomes of high diversity such as fast evolving microbes. Besides, these hybridization-based methods generally require more than one microgram of input DNA and therefore often require pre-amplification.

Here we present a method for obtaining metagenomic profiles of *H. pylori* microdissected from old FFPE samples. In addition to a laser capture microdissection (LCM) step, the method is based on a limited number of cycles of DNA pre-amplification and a modified sequencing protocol for the Roche 454 platform [5] using customized barcoded Y adaptors [6].

## Materials and Methods

### Ethics Statement

The study subjects provided informed consent. The study was approved by the ethics committee of the Medical Faculty, Uppsala University (Uppsala, Sweden).

We investigated two FFPE biopsies (FFPE 1 and FFPE 2), taken at gastroscopies in 1995 from two patients diagnosed with gastric cancer. The patients were enrolled in a previous endoscopy clinic-based study [7]. In addition to these biopsies, which were taken for morphological evaluation of the noncancerous gastric corpus mucosa (including semi-quantitative evaluation of *H. pylori* presence), biopsies from the same area were homogenized and frozen. The latter biopsies were stored at –80°C until the present experiment. Both patients were classified as having mild *H. pylori* infection (‘+’) by Hematoxylin & Eosin and Warthin–Starry silver staining. We chose patients with mild infection because the future application of our method is likely to involve subjects not only with heavy infection, but also with sparse presence of *H. pylori*. All new sequence data has been deposited in GenBank (Sequence Read Archive, Accession #: SRA024593.1).

### Immunohistochemisty (IHC)

**Figure 1:**
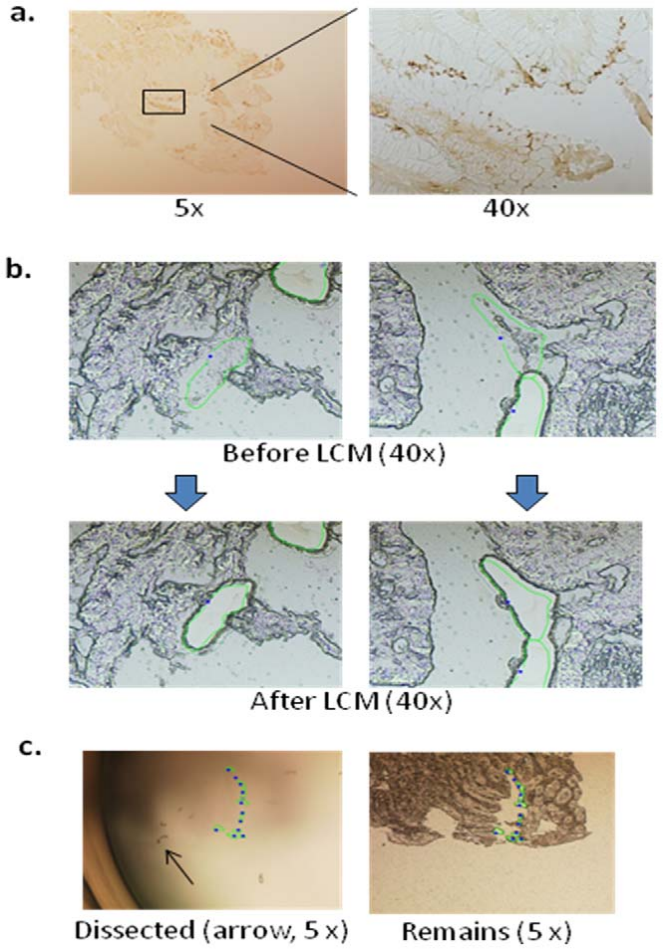
Laser capture microdissection a) Immunohistochemistry identified *H. pylori* colonizing area; b) Microdissection using neighboring sections; c) Dissected samples and remains.

From each of the FFPE biopsy blocks, six sections, each 5 µm thick, were cut with disposable and autoclaved microtome blade. The surface section was discarded to reduce contamination. The other 5 sections were placed on autoclaved de-ionized warm (about 45°C) water. To guide the microdissection of *H. pylori* on the adjacent de-paraffinated sections (Figure 1), the second and sixth sections were collected on glass slides for IHC staining of *H. pylori*. The IHC staining included a de-paraffination step by 3 rinses in xylene solution for 10 minutes each and re-hydration through sequential rinses in ethanol (99% 5 minutes twice; 95% for 2 minutes; 70% for 2 minutes) and in water for 2 minutes. Antigen retrieval was achieved through heat treatment at 95°C for 20 minutes in sodium-citrate buffer (pH 6.0), followed by non-specific background blocking in a solution containing 2% swine serum, 1% BSA and 0.1% azide in TBS pH 7.6 for 30 minutes. Primary antibody (polyclonal rabbit anti-*H. pylori*, DAKO, Denmark) 1∶1000 in blocking buffer was applied at ambient temperature (22°C) for 1 hour, then rinsed 3 times in fresh 1 x TBS, 0.5% Tween 20, each for 5 minutes. Endogenous peroxidase was blocked in 3% H_2_O_2_ for 10 minutes and rinsed in fresh 1 x TBS for 5 minutes. Secondary antibody (Swine anti-rabbit, biotinylated, DAKO) 1∶500 in 1 x TBS was applied and incubated at ambient temperature for 1 hour, and rinsed 3 times in fresh 1 x TBS, 0.5% Tween 20, each for 5 minutes. The sections were then incubated in strepavidin-biotin-peroxidase complex (DAKO) according to the manufacturer's instruction. The peroxidase was then developed by the 3,3′-diaminobenzidine tetrahydrochloride (DAB, Zymed), resulting in brown stained *H. pylori*.

### Laser capture microdissection (LCM)

The third to fifth sections were collected on molecular grade membrane-mounted PALM slides (Carl Zeiss). These sections were de-paraffinated in fresh xylene and rinsed in falling concentrations of ethanol as above, followed by laser capture microdissection (Carl Zeiss PALM System) of *H. pylori*, which was located under the guidance of the IHC staining results from above.

### DNA extraction of LCM samples

LCM samples were subjected to DNA extraction directly (without change of tubes) by adding extraction buffer (20 mM Tris, 0.1 mM EDTA, 0.5% Igepal, 1% Proteinase K fresh from stock) and incubated on a thermocycler at 60°C for 72 hours with heat cover on. The DNAs were purified with AMPure beads (180% volume, Agencourt) and eluted in 1 x TE buffer. Whole genome amplification was performed using the Sigma WGA4 kit according to the manufacturer's instruction, except that the procedure was started after the heat fragmentation step, because the LCM DNAs were already highly degraded, and that only 14 cycles of amplification were run instead of 25. Small aliquots of the amplified products were analyzed on 1% agar gel and no visible product was observed, as intended. The amplified products were purified by AMPure beads as above and followed by our modified 454 library preparation and sequencing method, as described previously [5].

### Re-culturing *H. pylori* and DNA extraction

For comparison, homogenized frozen biopsies taken during the same gastroscopy sessions and from the same anatomic site as the studied FFPE biopsies (all from the corpus) were thawed for re-culturing of *H. pylori* under standard condition. The re-cultured isolate sweeps (containing hundreds of colonies) were collected in PBS and used for DNA extraction using Qiagen DNeasy Kit according to the manufacturer's instruction, followed by DNA nebulization, modified library preparation and 454 sequencing.

### Data Analysis

Sequence data of culture *H. pylori* DNA were assembled first using the Roche gsAssembler v2.5.3 with default parameters except that the stringency of alignment identity was increased from default 90% to 95%, as the identity is normally higher than 95% for defining species, although there is no consensus of a definite level. Since these isolate sweeps potentially contained different strain genotypes, the assembly resulted in many short contigs. These contigs were used as references for alignment of sequences from FFPE samples.

Because the FFPE samples were amplified before sequencing, we identified a set of Sigma WGA primers by mock gsAssembler and trimmed away in further analyses. FFPE DNA sequences were aligned with GenBank reference genomes (all ten available at the time of this analysis), each separately as well as combined, and with the assembled contigs from the culture isolates DNA using Roche GS Mapper (v2.5.3), with default level of minimum overlap 40 nt and a minimum identity of 90%. ANOVA was used to compare the differences of mapped length and identity. Venn diagrams were used to illustrate the numbers of FFPE sequence reads that mapped to the ten reference genomes as combined, and to assembled contigs from the two culture samples. Fisher Exact test was used to compare frequencies in the Venn diagrams.

To annotate the FFPE sequences that were mapped only to the assembled contigs of the culture from the corresponding patient, these sequences were aligned to the ‘nr’ database (All non-redundant GenBank CDS translations + PDB + SwissProt + PIR + PRF, excluding environmental samples from WGS projects) using the BLASTx web application (http://blast.ncbi.nlm.nih.gov/Blast.cgi) with default parameters, and the annotation features were extracted.

To assess the degree of amplification bias, we aligned the FFPE sequences to the assembled contigs from the culture samples (all contigs larger than 100 bp lined up by their sizes) from the same patients and the ten GenBank *H. pylori* strains. Besides mapping at single-base resolution, we also used a crude resolution as a proxy for genes for the purpose of epidemiological studies on presence and absence of genes. This crude resolution was a 1000-bp bin along all the assembled contigs, or the open reading frames in GenBank strains. The reason for using a bin size of 1000-bp was because the average size of *H. pylori* genes is about 1000 base pairs.

## Results

### Alignments of FFPE *H. pylori* sequences

**Figure 2:**
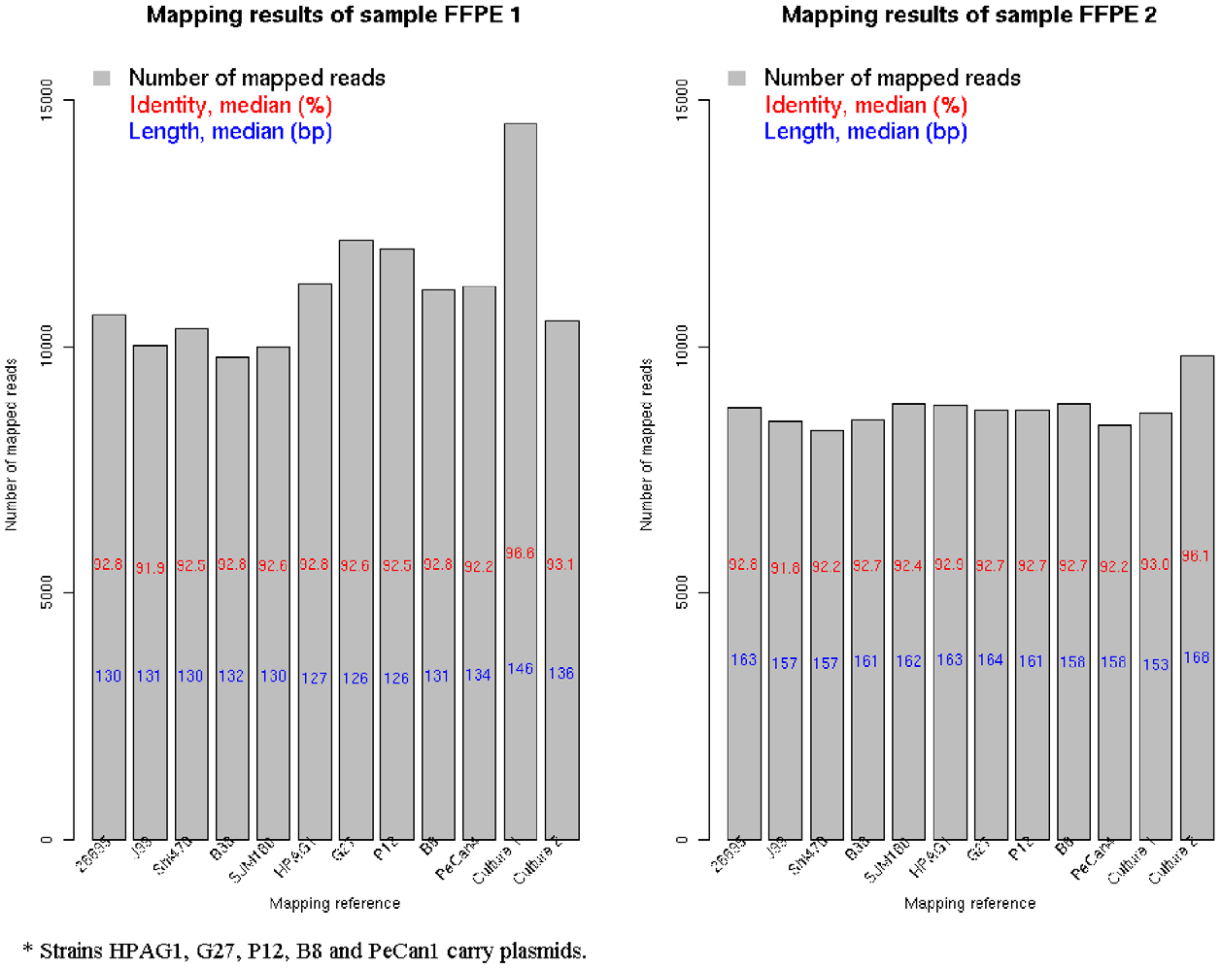
Alignment identity, length and number of read of two FFPE samples against each of the ten GenBank *H. pylori* reference genomes and the culture samples of the two patients, respectively.

In total, 241,126 and 201,709 sequence reads were obtained from the two FFPE samples, respectively. Among them, 14,532 (6.0%) and 9,830 (4.9%) could be mapped to contigs assembled from DNA of cultured *H. pylori* from the same patients (Culture 1 and Culture 2; Figure 2). When aligning FFPE sequences with each of the ten GenBank reference genomes, the numbers of aligned reads were only ∼11,000 and ∼8,700, respectively. The reference strains HPAG1, G27, P12, B8 and PeCan4 contain plasmids. Alignment of FFPE 1 sequences with these five strains resulted in on average 1,395 (12.1%) higher number of reads than with the five strains that lack plasmids (Figure 2). Alignment of raw sequences from Culture 1 also resulted in a greater (7.7%) number of reads matching to plasmid containing reference strains. No such difference was observed for sample FFPE 2 or Culture 2.

Alignment identity of FFPE 1 and Culture 1 was 96.6%, and FFPE 2 and Culture 2 96.1% (Figure 2). Cross FFPE-culture alignment of the two patients gave an identity of 93.1% for FFPE 1 and Culture 2, and 93.0% for FFPE 2 and Culture 1. Alignments of FFPE samples with each of the ten reference strains (tenRef) showed similar identity as the cross FFPE-culture alignment (range 91.8% to 92.9%). Alignment identity of raw culture sequences with assembled culture sequences was 98.7% for Culture 1, and 98.8% for Culture 2 (data not shown). Alignment length of FFPE 1 was longer (median 146 bp) with Culture 1 than with others (medians ranged 127 – 134 bp), and similarly, the median alignment length for FFPE 2 with Culture 2 (168 bp) exceeded that with others (medians ranged 157 – 164 bp, Figure 2). Statistical analyses showed that the paired FFPE-Culture has longer reads and higher identity than other mappings (all P values were <10^−7^), whereas the FFPE1-Culture2 or FFPE2-Culture1 mapping have similar length or identity as some of the FFPEs-tenRef mappings (some P values were >0.05, data not shown).

### FFPE Sequences aligned only to the culture sequences from the same patient

**Figure 3:**
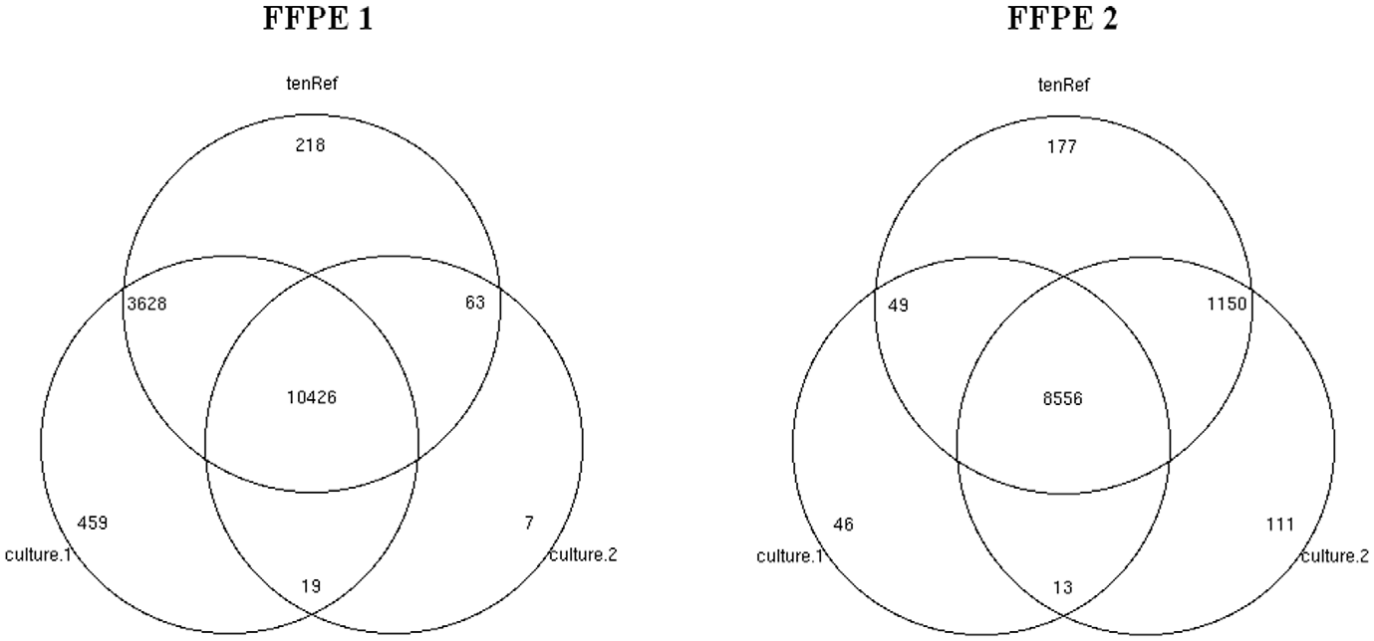
Venn diagram showing the numbers of mapped reads from two FFPE samples against the ten *H. pylori* references (tenRef) and the culture samples of the two patients.

Venn diagrams (Figure 3) show that there were higher numbers of FFPE sequences aligned with the culture sequences from the same patient than with the ten reference genomes combined or with the culture sequences from the other patient. In addition, the number of FFPE 1 reads that mapped uniquely to Culture 1 was higher than the numbers of FFPE 1 reads that mapped uniquely to tenRef or with Culture 2 (both P values <10^−16^). The number of FFPE 2 reads that mapped uniquely to Culture 2 was higher than the number of FFPE 2 mapped uniquely to Culture 1, but less than the tenRef (both P values <10^−4^). Annotation of the FFPE sequences aligning only with culture sequences from the same patient revealed an abundance of genes that are components of plasmid (relaxase, transposase, replicase, etc.) and of restriction-modification systems (see supplementary files File S1 and File S2.).

### Whole genome amplification and assessment of its bias

**Figure 4:**
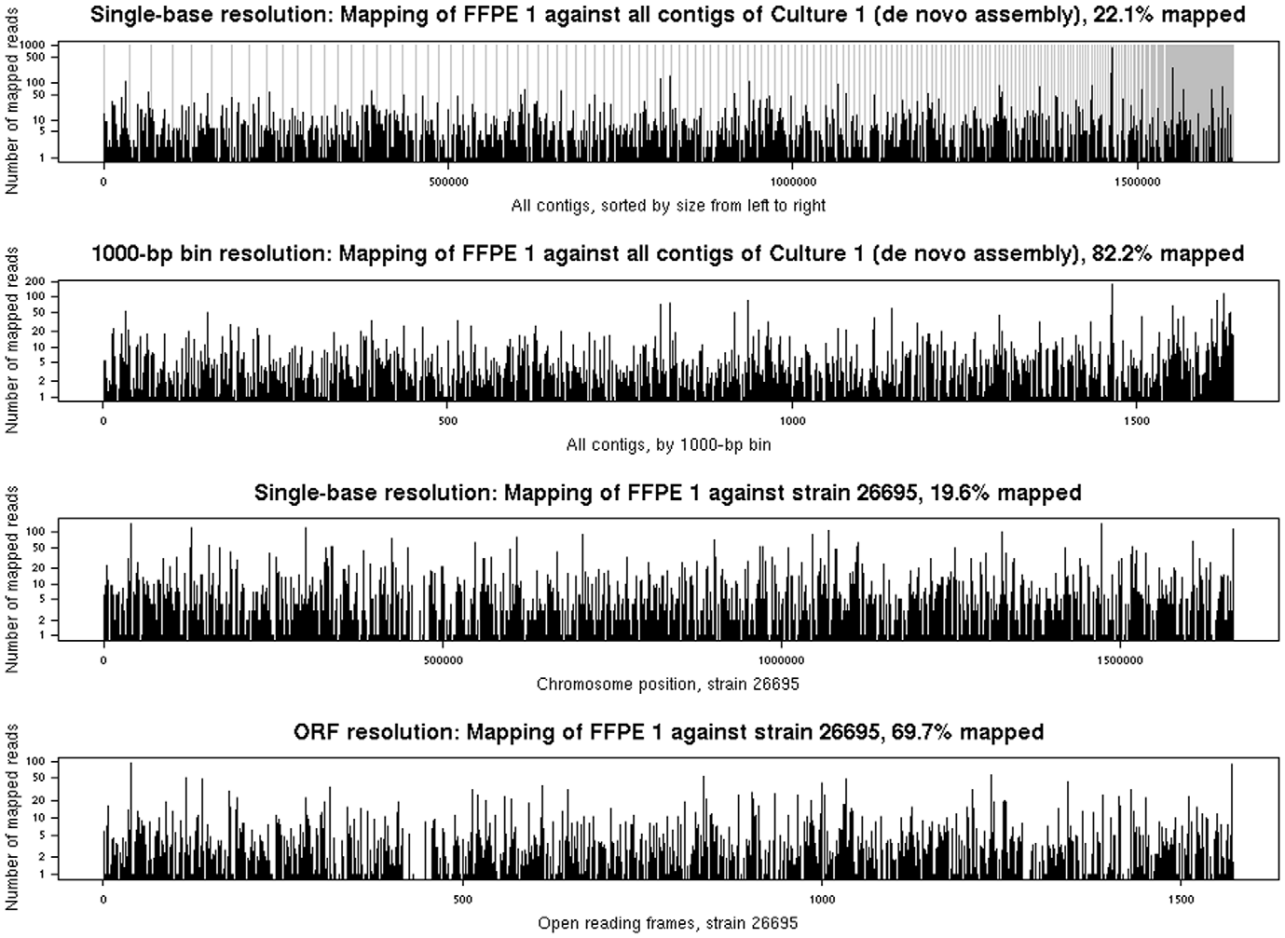
Sequence distributions of FFPE 1 across all contigs of Culture 1 (de novo assembly) at resolutions of single-base and 1000-bp bin, and across the chromosome of strain 26695 at resolutions of single-base and open reading frame (ORF).

Mapping of the FFPE sequences onto the assembled contigs or onto the ten reference genomes showed that the FFPE sequences were fairly evenly distributed (Figure 4 and supplementary Figures S1 and S2). The FFPE 1 sequences covered 22.1% of all Culture 1 assembled contigs at single-base resolution, and 82.2% at 1000-bp bin resolution. When mapping onto the strain 26695 genome, the corresponding percentages were 19.6% and 69.7% (Figure 4); and for strain B8 19.2% and 66.9% (plasmid 63.8% and 100%); B38 19.4% and 72.1%; G27 19.7% and 72% (plasmid 72.1% and 100%); HPAG1 19.8% and 71.8% (plasmid 72.1% and 100%); J99 18.6% and 70.2%; P12 19.2% and 69.8% (plasmid 66.4% and 90%); PeCan4 18.9% and 68.7% (plasmid 37.3% and 75%); Shi470 19.4% and 68.2%; and SJM180 18.9% and 68.3% (Supplementary Figure S1). For FFPE 2 sequences, apart from lower mapping percentages (Culture 2 12.6% and 60.1%, GenBank strains about 11% and 50%), a major difference compared with FFPE1 results was that there was nearly no sequence mapped to the plasmids (see details in Supplementary Figure S2). GC content is one of the main factors influencing amplification bias [3]. The GC content of amplified FFPE *H. pylori* sequences in sample FFPE 1 and FFPE 2 was 40.06% and 40.99%, respectively, close to the range of 38.89% to 39.19% of reference *H. pylori* genomes (UCSC genome browser).

### Sequencing depth versus number of unique bins covered

**Figure 5:**
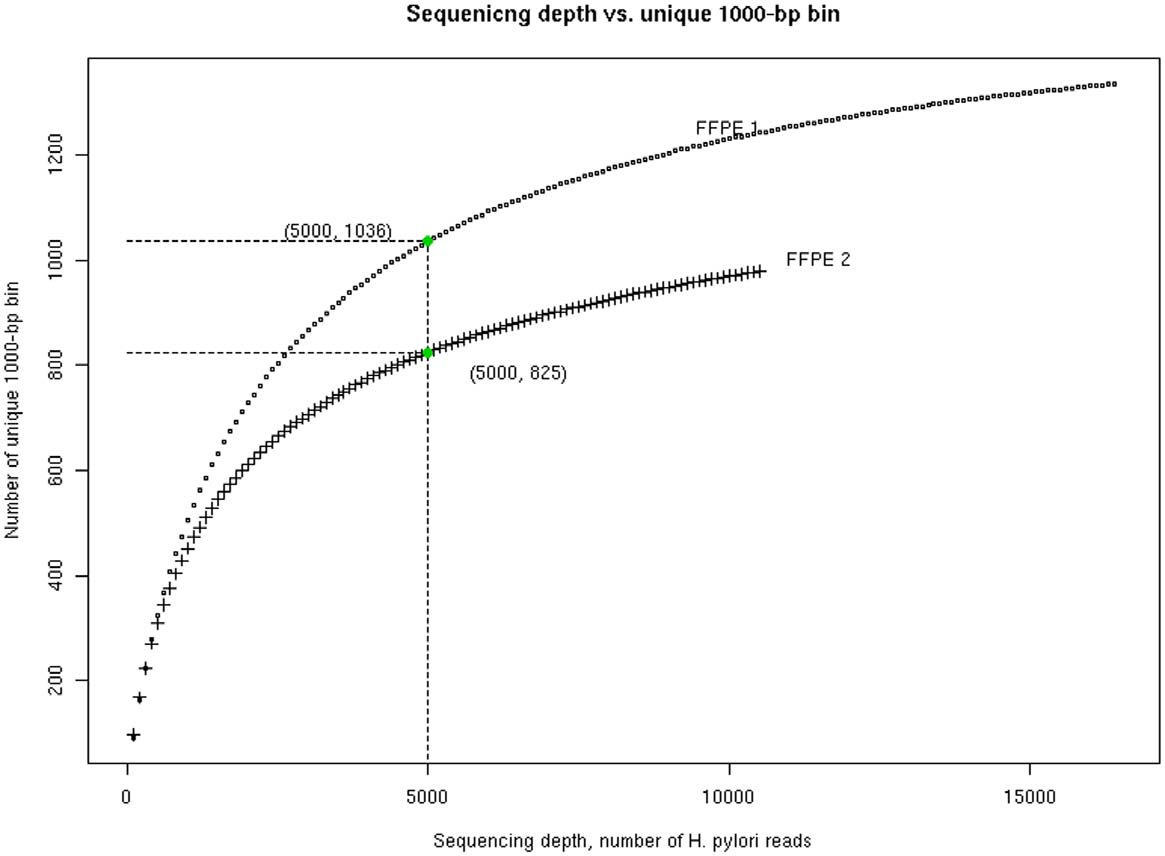
Simulations of sequencing depth versus yield of 1000-bp bins for the two laser capture microdissected samples (FFPE 1 and FFPE 2). Each data point was obtained through 1000 simulations, with replacement, where each simulation contained a specified number (the X axis) of randomly sampled sequences, increased in steps of 100 up to the total number of sequences available. The number of unique bins for each simulation dataset was calculated, as was the average number derived from 1000 simulation datasets. These average numbers were plotted. The two data points labeled where the curves start to level off approximately, as examples of reasonable sequencing depth for cost-effective consideration.

Figure 5 shows the relation between sequencing depth and yield of unique 1000-bp bins, assessed for each FFPE sample through simulations. The yield of unique bins increased with increasing sequencing depth, but around 5,000 sequence reads it approached a plateau, similarly for both of the samples.

## Discussion

Higher identity was observed when aligning FFPE metagenomic samples with the culture sample from the same host (∼96.4%) than with the reference genomes or with the culture sample of the other patient (∼92.5%). This demonstrates that the metagenomic profiles of *H. pylori* dissected from 15 year old FFPE sections faithfully represent the *H. pylori* populations of the patients. We observed an identity of about 98.7% when aligning culture raw sequences with culture assembled contigs from the same patient. However, higher than 99% identity is expected when the same *H. pylori* strain is compared using 454 sequencing and GS mapping software, as we have shown previously [5]. The lower identity suggests the presence of multiple strain genotypes in the isolate sweeps. The even lower identity (∼2.3% lower) when comparing FFPE reads with culture assembled contigs may be due to error prone translesion synthesis during PCR using formalin-fixed DNA [8]. However, given an estimated translesion synthesis error rate of only 0.1% per base [8], other conceivable explanations might be i) a patchy distribution of *H. pylori* in the stomach leading to different strains of *H. pylori* being sampled in the FFPE biopsy and in the culture biopsy; ii) the existence of viable but nonculturable coccoid and/or degenerative forms [9], [10] of *H. pylori in vivo* which could only be sequenced with metagenomic methods.

Specificity of enriched sequences, i.e. the fraction of sequences aligning to target sequences, is one of the main parameters in evaluating enrichment performance. About 5% of DNA fragments that we dissected from the FFPE sections could be mapped to *H. pylori*, and the rest was mostly human (82.6% in FFPE 1 and 64.0% in FFPE 2; data not shown) and unknown (no hits, 11.7% in FFPE1 and 27.7% in FFPE2). Considering the size of the human genome (∼3.3 billion base pairs) and that of *H. pylori* (∼1.6 million base pairs), the 5% being *H. pylori* reflected a contamination of 1 human cell in more than one hundred *H. pylori* cells, demonstrating a significant enrichment of *H. pylori* considering that a biopsy typically contains much more than 1% human tissue cells. In a recent study, laser capture microdissection was applied for analyzing microbial communities in two fresh biopsies from human colon [11]. The results suggested that microbial DNA could be cleanly separated from host DNA. However, total avoidance of any contamination is implausible; human DNA is likely released during biopsy sectioning and spreads in the downstream washing steps. Unfortunately, the percentage human DNA reads over total reads was not reported in the publication [11]. The human DNA observed in our dissected material might partly derive from tissue cell disruption during long-term storage of the biopsy at room temperature and mechanical trauma during sectioning.

An alternative to microdissection may be a hybridization-based method, which could be used for enrichment-and-sequencing or for direct detection. This approach has been widely used, e.g. for exon sequencing. However, hybridization methods rely on current and thus probably incomplete information on the genomic makeup of the studied organism, which may be a limitation especially in the enrichment of highly diverse microbial genomes. Also, hybridization relies on good sequence matching and good hybridization conditions (buffer, temperature, time, etc.) to minimize false hybridizations and missed targets. Our finding of sequences shared between the FFPE and culture of the same patient but not aligning with the comparably large selection of reference genomes demonstrated that even in an imaginary perfect hybridization with probes covering the entire genome of all ten references strains, some true but highly variant *H. pylori* sequences would still be missed. The omission of true *H. pylori* reads is not surprising as *H. pylori* is known to be highly diverse [12]. One question that might arise concerns how to identify these missed true sequences when, in reality, re-cultures are unavailable in most of the cases. By using our proposed metagenomic approach, functional genes displaying significant difference between cases and controls can be determined using e.g. BLASTx against the “nr” database followed by comparisons of their frequencies in the two groups, without the need for closely matching reference genomes.

The percentage of target detected is another important parameter. This percentage increases with increasing sequencing depth and levels off at a certain point beyond which little is gained by additional data. In this proof-of-principle study, we devoted a sequencing capacity of 200,000 reads to each FFPE sample. The sequencing cost can be reduced by reducing sequencing depth to the level where the sequencing depth versus gene yield curve levels off and the marginal cost per additional gene becomes prohibitive. Thus, more samples, such as in an epidemiological setting, could be sequenced with the same budgetary constraint. In our study samples, the number of unique bins (proxy for genes) obtained started to level off at round 5,000 reads, yielding about 1,000 unique bins which corresponding to about 65% of the number of genes in a typical *H. pylori* genome. Foreseeably, sequencing redundancy is rapidly becoming less of a concern as the sequencing cost continues to fall.

The amount of input DNA required in subsequent steps is also critical for the choice of enrichment method. The three commercial hybridization-based methods all require input DNA quantities above one microgram [4], sometimes necessitating pre-amplification. In the previous study using microdissection to analyze microbes from fresh colon biopsies [11], about three nanogram of DNA was dissected from ten 0.8- µm thick sections and was amplified by a multiple displacement amplification method, which could have been unnecessary if the library preparation protocol had been adjusted for nanogram input DNA with the capability to yield tens of millions of reads. Our DNA yield was much less (not detectable by a fluorometry method) due to moderate *H. pylori* growth, thinner sections, and degraded DNA and still requires pre-amplification. Whole genome amplification always introduces some bias, varying with the amplification method [3], with the multiple displacement amplification method giving less bias than others. However, as multiple displacement amplification requires fragment longer than 500 bp (http://www.qiagen.com/products/repli-gffpekit.aspx#Tabs=t1, accessed Mar 15, 2011), its application on decade-preserved FFPE biopsies is limited. We therefore applied a PCR-based method and amplified for 14 cycles (instead of the recommended 25) to reduce bias. GC content is the most established risk factor for amplification bias in PCR-based methods [3]. We observed similar GC contents in the amplified and sequenced *H. pylori* reads (40% and 41%) as compared with the reference genomes (∼39%). Further, the fairly evenly distributed alignment of amplified FFPE *H. pylori* sequences to the reference *H. pylori* genome demonstrated that the amplification bias was minor, although not totally absent.

In all, physical separation through microdissection followed by sequencing provides an appealing alternative to commercial hybridization-based methods when microbial DNA needs to be separated from host DNA, with an additional advantage of being able to capture novel and highly variant genes. Application of this method on FFPE material from the distant past might hopefully unveil markers of a carcinogenic potential in *H. pylori* strains colonizing the stomach before irreversible damage has occurred, and thus pave the way for targeted chemoprevention.

## Supporting Information

Figure S1 Mapping results of FFPE 1 sequences with nine GenBank *H. pylori* strains.
(PDF)

Figure S2 Mapping results of FFPE 2 sequences with Culture 2 and ten GenBank *H. pylori* strains.
(PDF)

File S1 Blastx results of those FFPE 1 sequences aligned only with Culture 1 sequences.
(DOC)

File S2 Blastx results of those FFPE 2 sequences aligned only with Culture 2 sequences.
(DOC)
