# Supplementary material for: A Method for Metagenomics of Helicobacter pylori from Archived Formalin-Fixed Gastric Biopsies Permitting Longitudinal Studies of Carcinogenic Risk
Source: PLoS One. 2011 Oct 21;6(10):e26442. doi: 10.1371/journal.pone.0026442 (PMC3198776; doi:10.1371/journal.pone.0026442)
Supplement: Figure S1 — Mapping results of FFPE 1 sequences with nine GenBank H. pylori strains. (PDF) [file pone.0026442.s001.pdf]

Supplementary Figure 1. Mapping results of FFPE1 sequences with nine GenBank *H. pylori* strains.

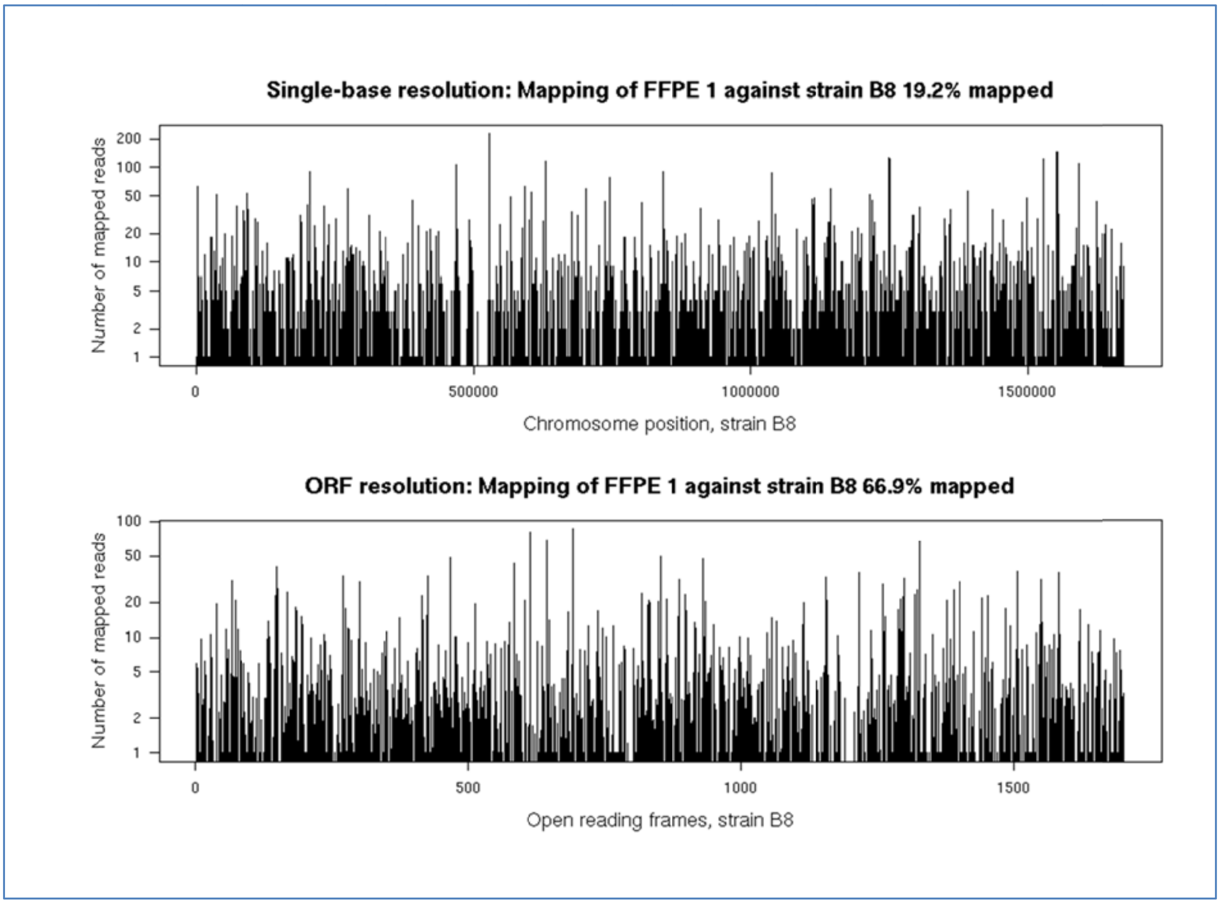

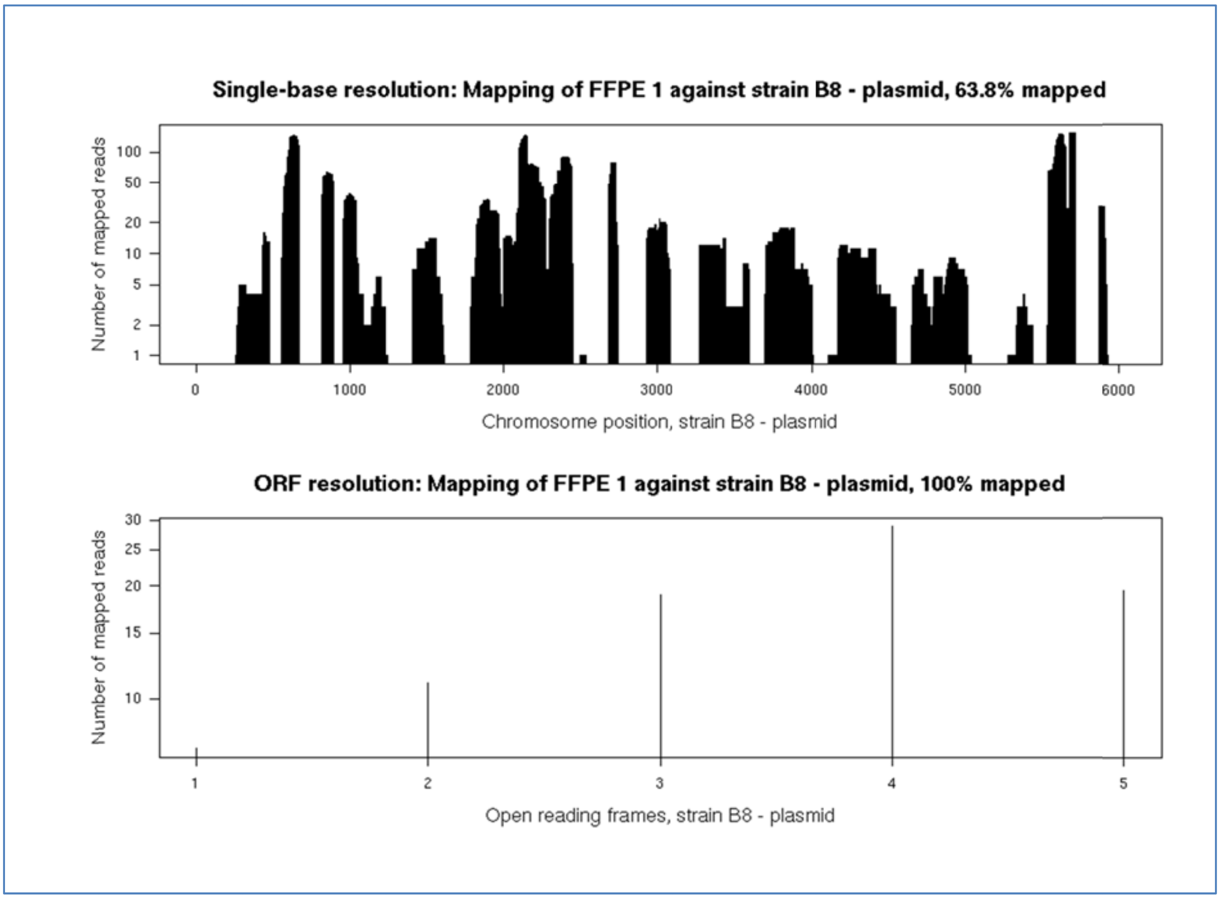

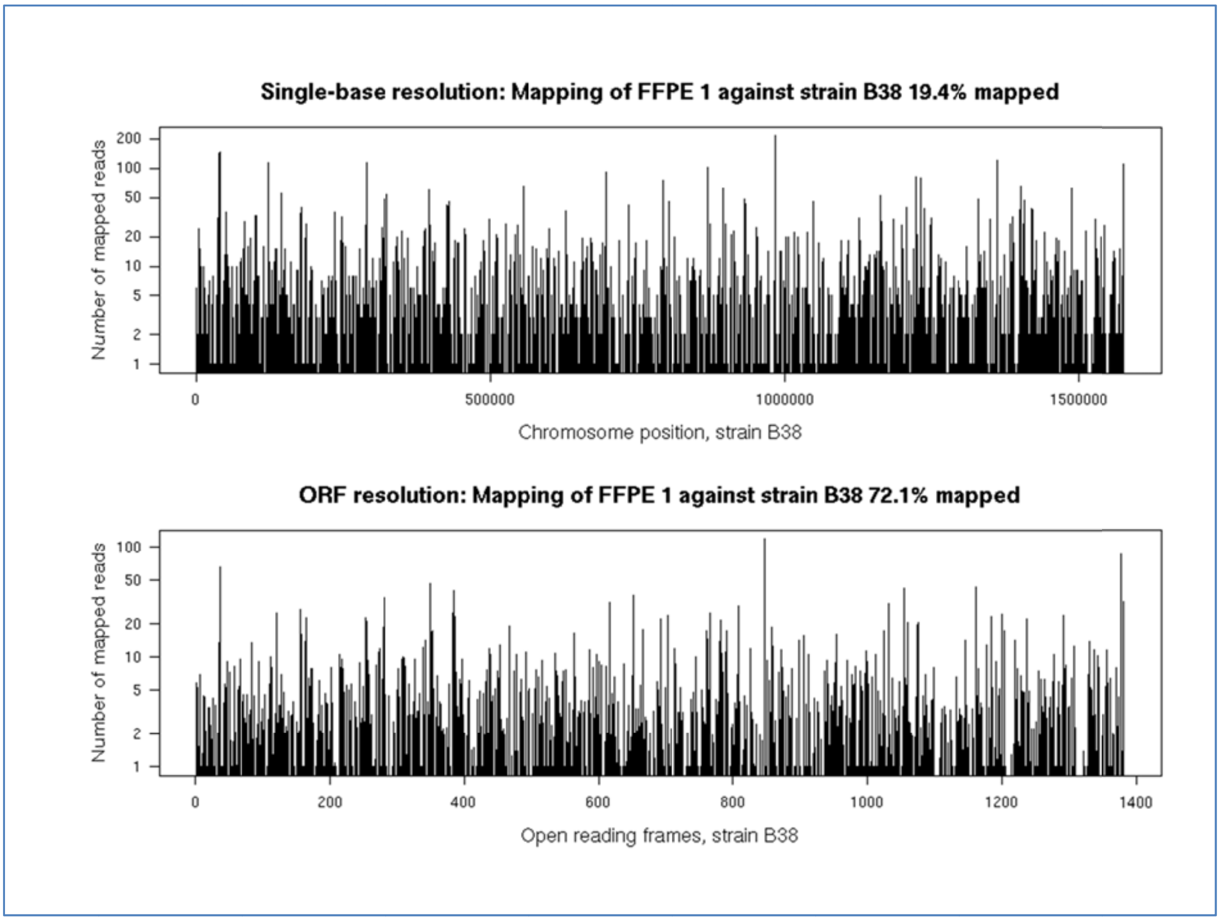
Single-base resolution: Mapping of FFPE 1 against strain B38 19.4% mapped
Number of mapped reads
Chromosome position, strain B38
ORF resolution: Mapping of FFPE 1 against strain B38 72.1% mapped
Number of mapped reads
Open reading frames, strain B38

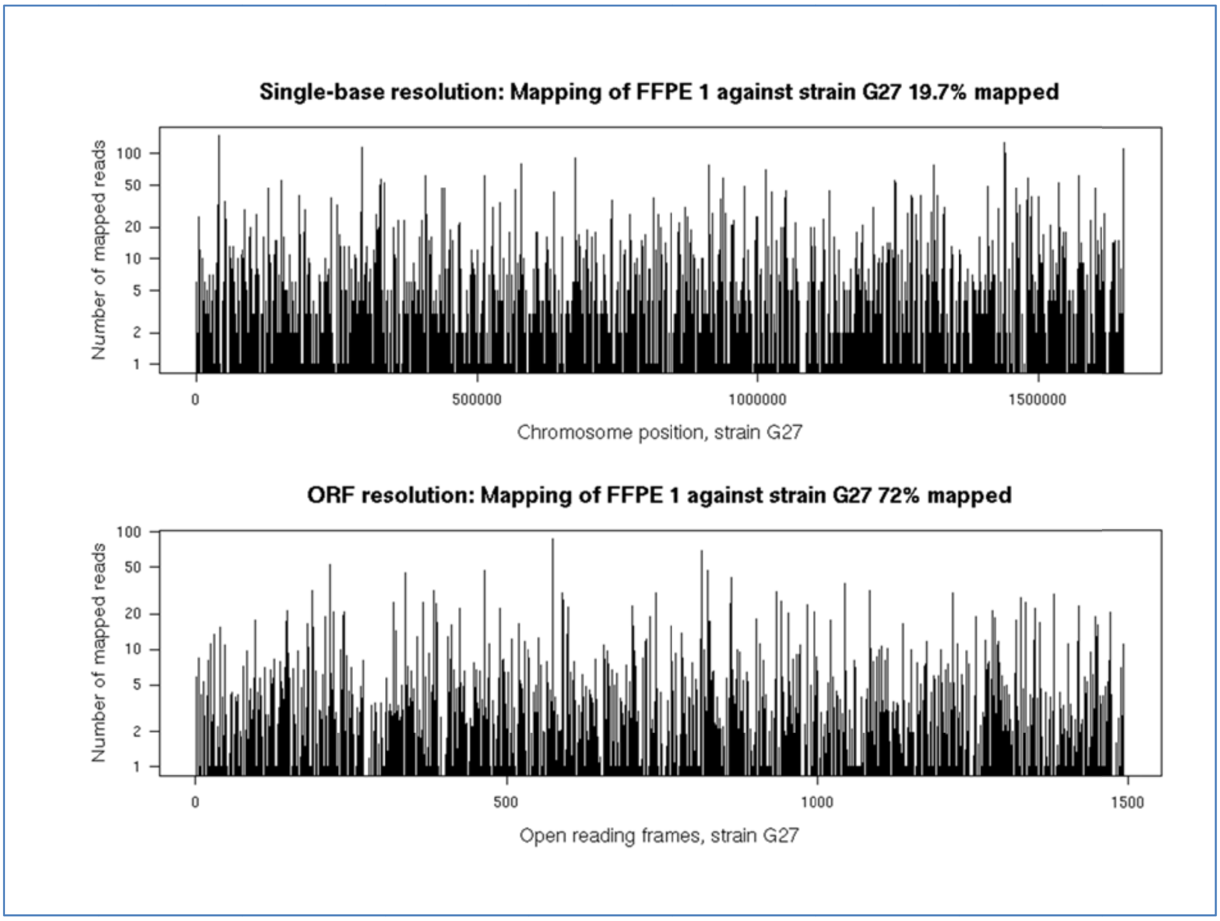
Single-base resolution: Mapping of FFPE 1 against strain G27 19.7% mapped
Number of mapped reads
Chromosome position, strain G27
ORF resolution: Mapping of FFPE 1 against strain G27 72% mapped
Number of mapped reads
Open reading frames, strain G27

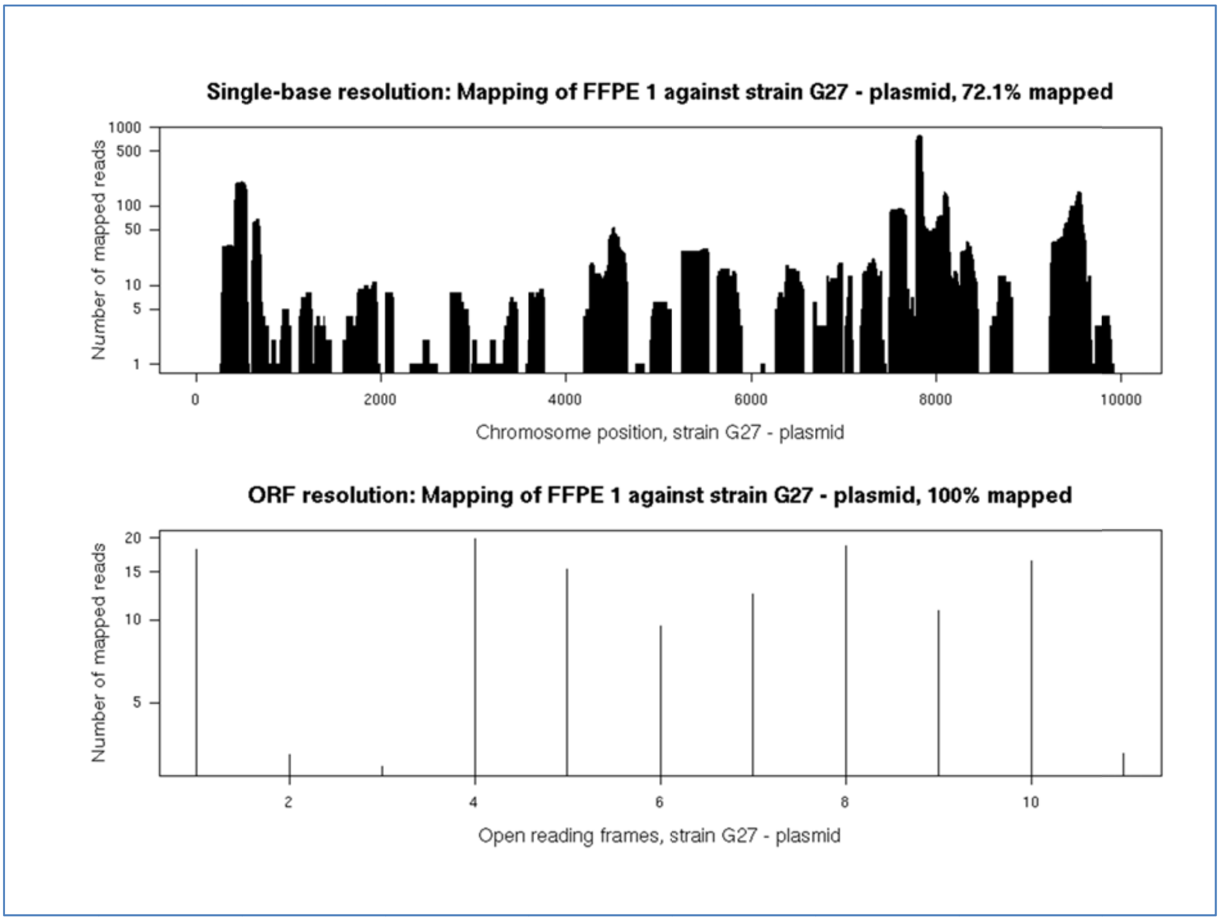

Single-base resolution: Mapping of FFPE 1 against strain G27 - plasmid, 72.1% mapped
Number of mapped reads
Chromosome position, strain G27 - plasmid
ORF resolution: Mapping of FFPE 1 against strain G27 - plasmid, 100% mapped
Number of mapped reads
Open reading frames, strain G27 - plasmid

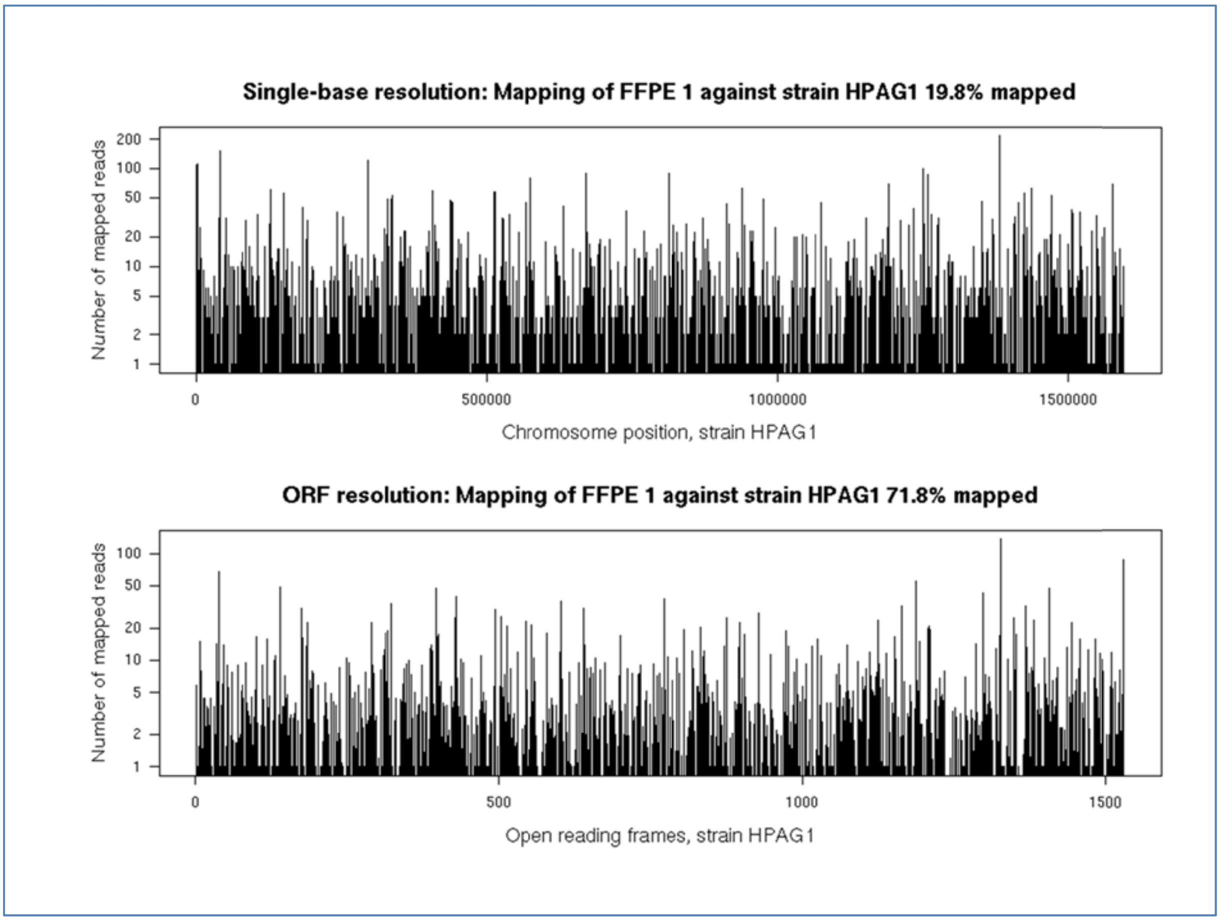

Single-base resolution: Mapping of FFPE 1 against strain HPAG1 19.8% mapped
Number of mapped reads
Chromosome position, strain HPAG1
ORF resolution: Mapping of FFPE 1 against strain HPAG1 71.8% mapped
Number of mapped reads
Open reading frames, strain HPAG1

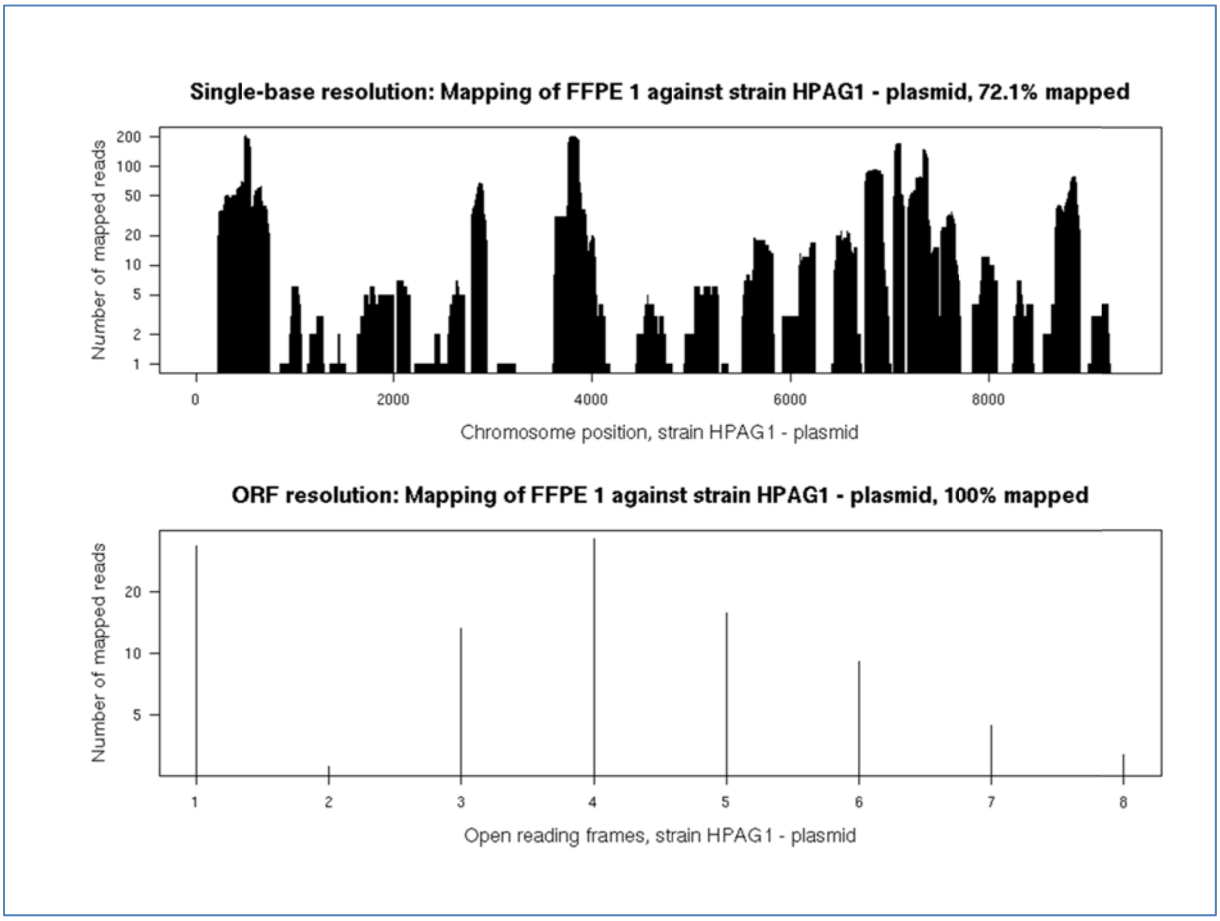
Single-base resolution: Mapping of FFPE 1 against strain HPAG1 - plasmid, 72.1% mapped
Number of mapped reads
Chromosome position, strain HPAG1 - plasmid
ORF resolution: Mapping of FFPE 1 against strain HPAG1 - plasmid, 100% mapped
Number of mapped reads
Open reading frames, strain HPAG1 - plasmid

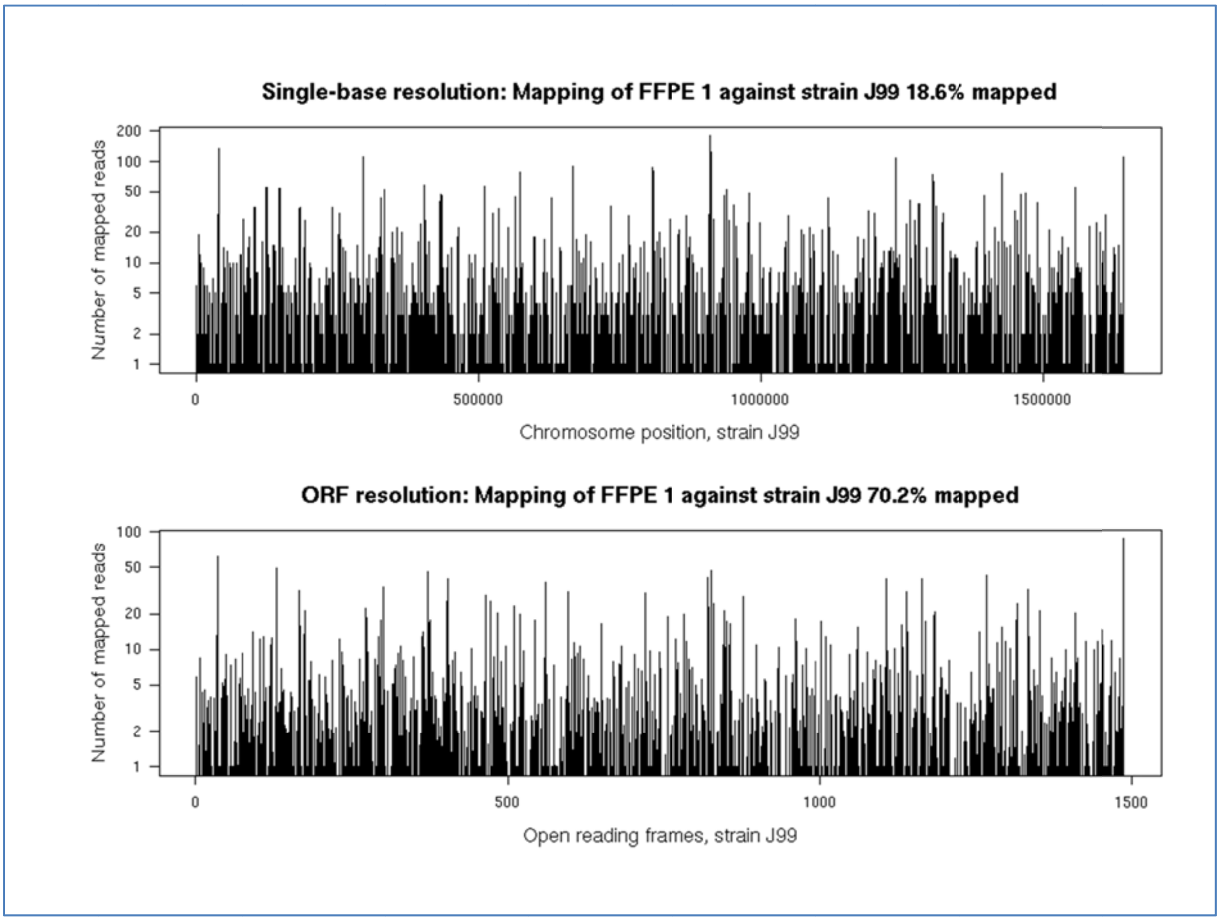
Single-base resolution: Mapping of FFPE 1 against strain J99 18.6% mapped
Number of mapped reads
Chromosome position, strain J99
ORF resolution: Mapping of FFPE 1 against strain J99 70.2% mapped
Number of mapped reads
Open reading frames, strain J99

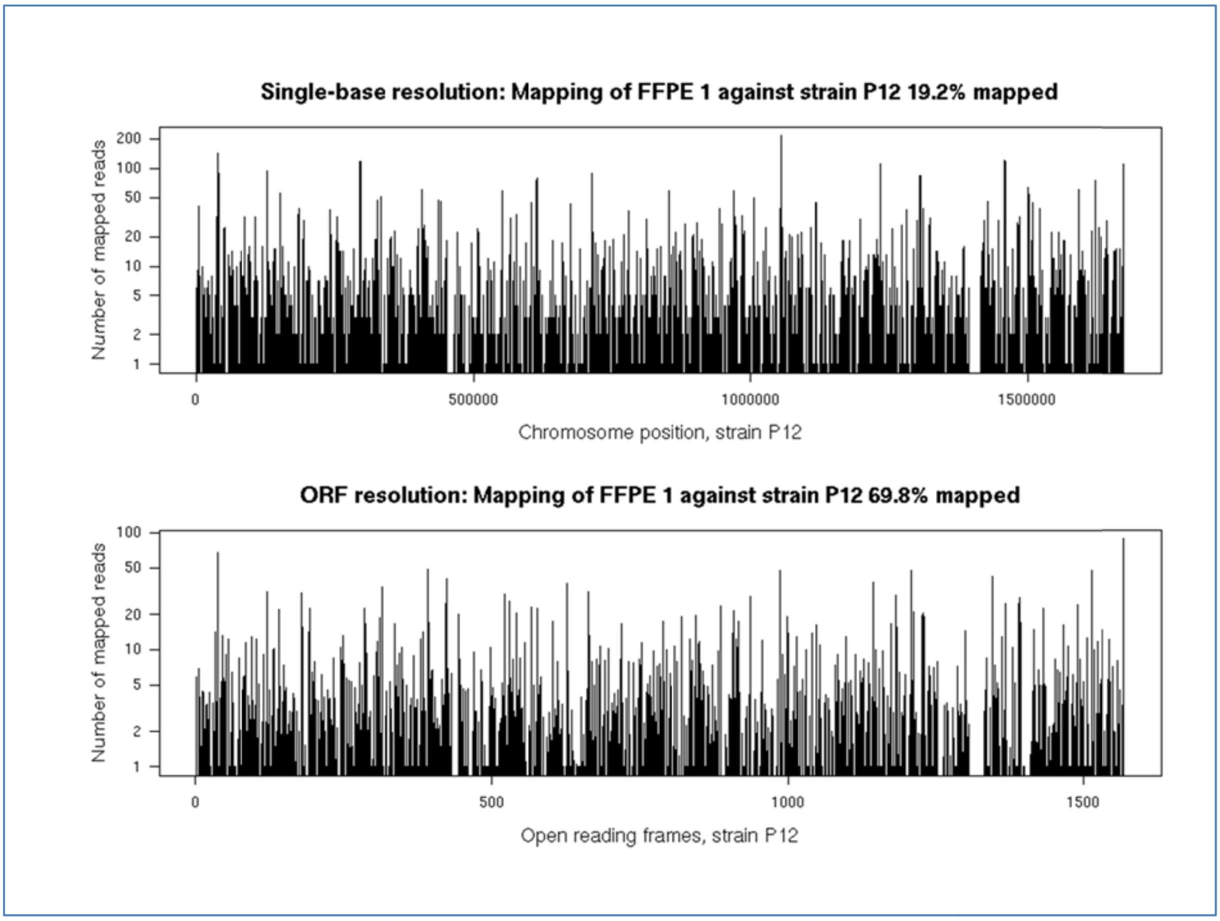

Single-base resolution: Mapping of FFPE 1 against strain P12 19.2% mapped
Number of mapped reads
Chromosome position, strain P12
ORF resolution: Mapping of FFPE 1 against strain P12 69.8% mapped
Number of mapped reads
Open reading frames, strain P12

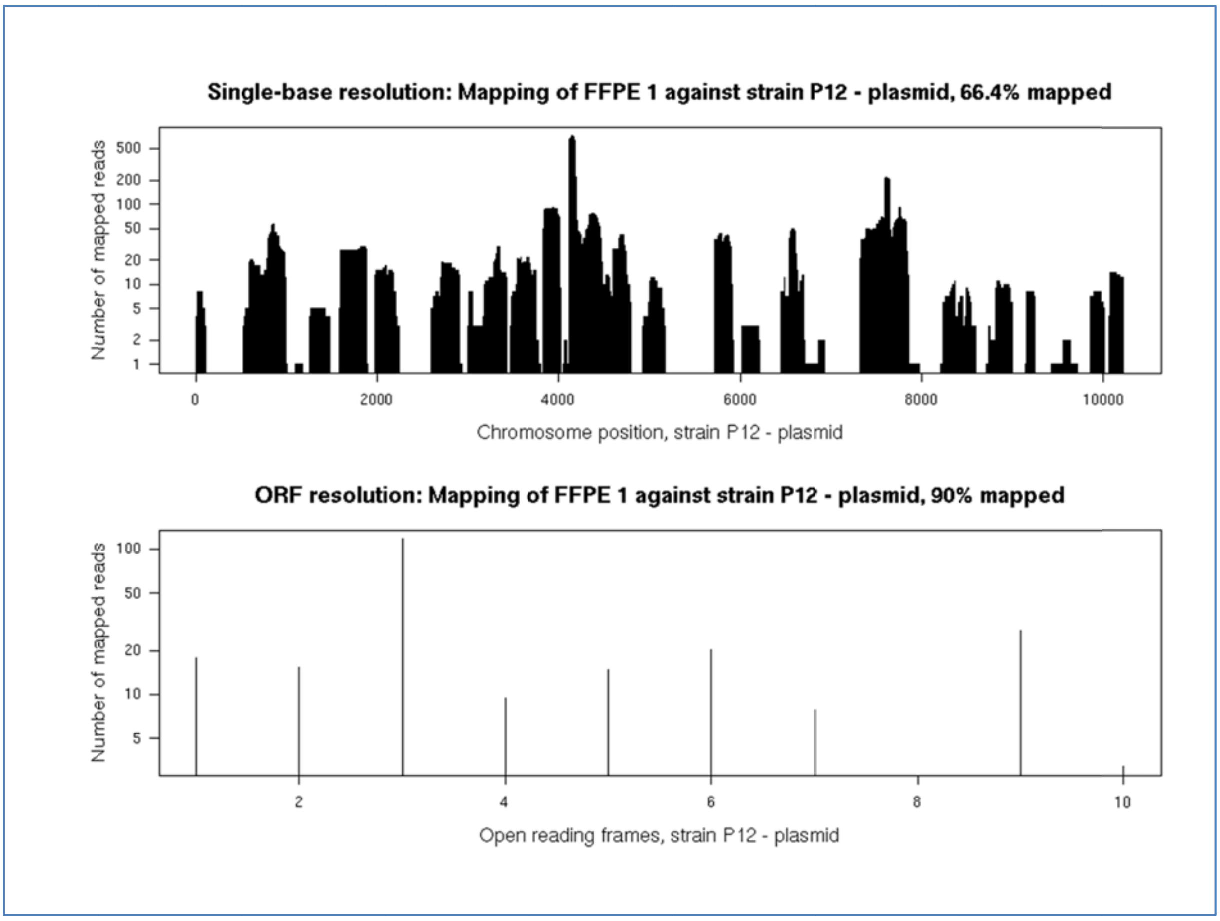

Single-base resolution: Mapping of FFPE 1 against strain P12 - plasmid, 66.4% mapped
Number of mapped reads
Chromosome position, strain P12 - plasmid
ORF resolution: Mapping of FFPE 1 against strain P12 - plasmid, 90% mapped
Number of mapped reads
Open reading frames, strain P12 - plasmid

Single-base resolution: Mapping of FFPE 1 against strain PeCan4 18.9% mapped

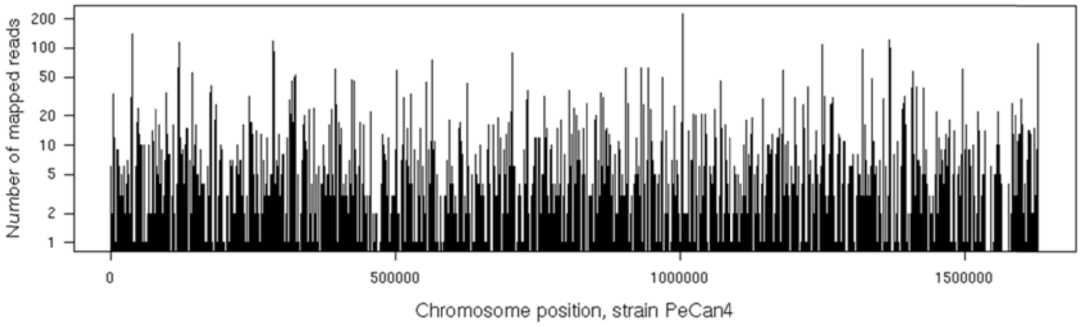

ORF resolution: Mapping of FFPE 1 against strain PeCan4 68.7% mapped

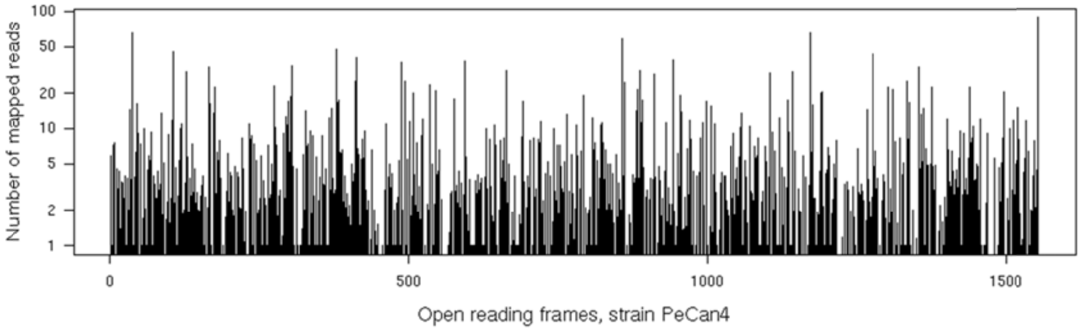

Single-base resolution: Mapping of FFPE 1 against strain PeCan4 - plasmid, 37.3% mapped

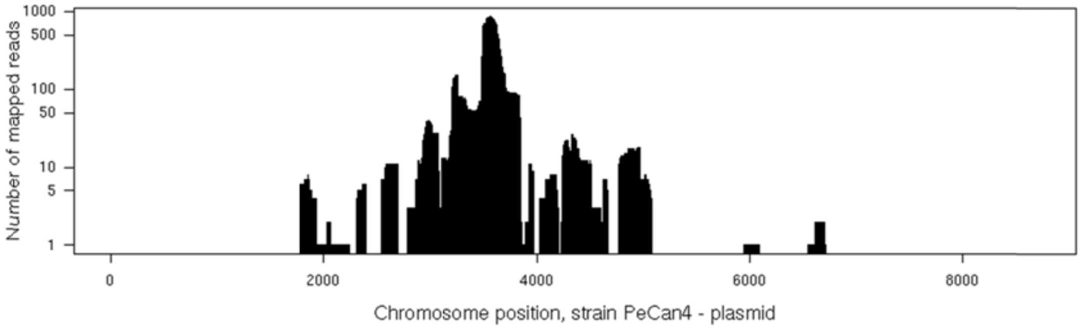

ORF resolution: Mapping of FFPE 1 against strain PeCan4 - plasmid, 75% mapped

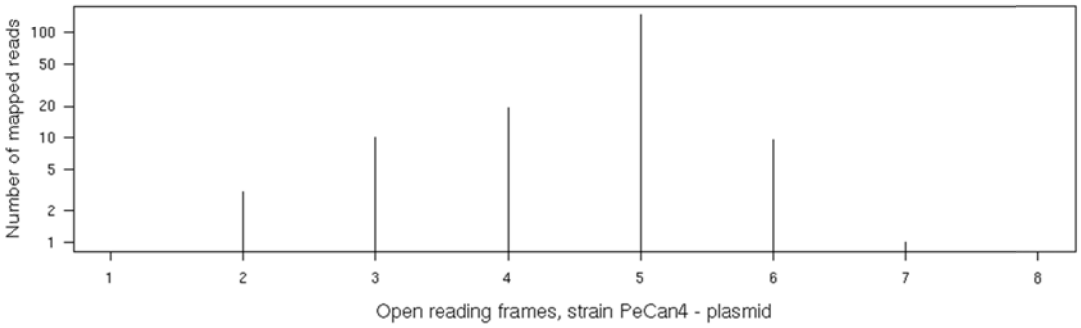

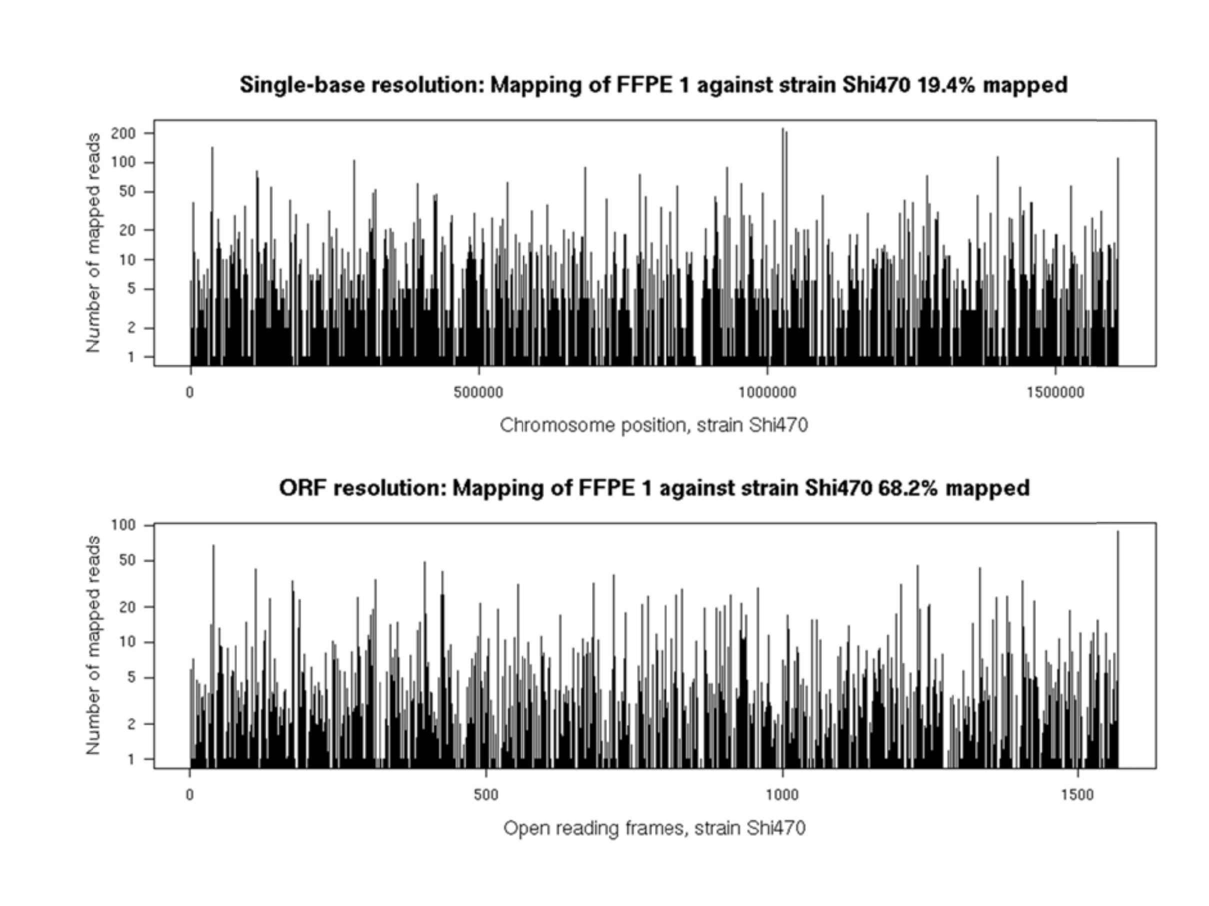
Single-base resolution: Mapping of FFPE 1 against strain Shi470 19.4% mapped
Number of mapped reads
200
100
50
20
10
5
2
1
0
500000
1000000
1500000
Chromosome position, strain Shi470
ORF resolution: Mapping of FFPE 1 against strain Shi470 68.2% mapped
Number of mapped reads
100
50
20
10
5
2
1
0
500
1000
1500
Open reading frames, strain Shi470

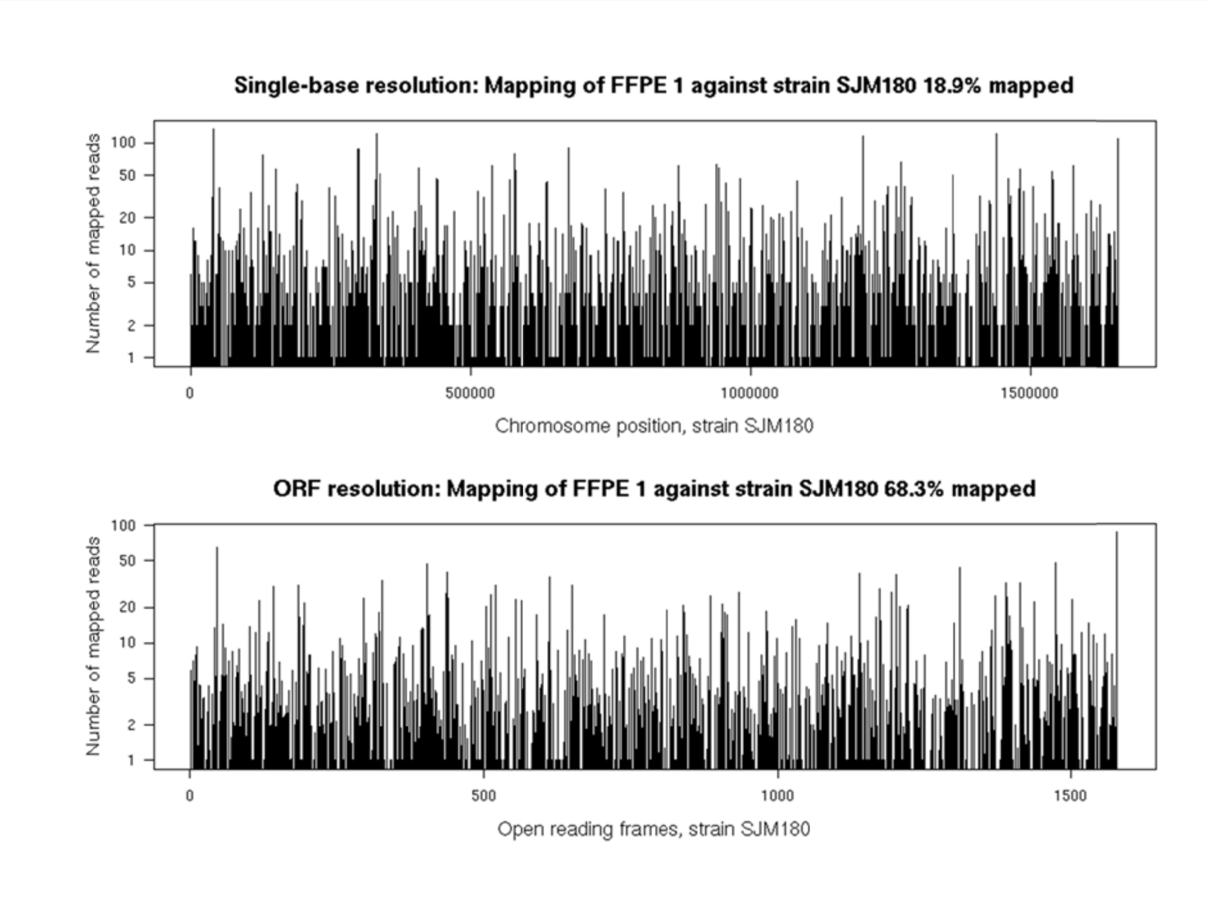
Single-base resolution: Mapping of FFPE 1 against strain SJM180 18.9% mapped
Number of mapped reads
100
50
20
10
5
2
1
0
500000
1000000
1500000
Chromosome position, strain SJM180
ORF resolution: Mapping of FFPE 1 against strain SJM180 68.3% mapped
Number of mapped reads
100
50
20
10
5
2
1
0
500
1000
1500
Open reading frames, strain SJM180
