# Supplementary material for: A Method for Metagenomics of Helicobacter pylori from Archived Formalin-Fixed Gastric Biopsies Permitting Longitudinal Studies of Carcinogenic Risk
Source: PLoS One. 2011 Oct 21;6(10):e26442. doi: 10.1371/journal.pone.0026442 (PMC3198776; doi:10.1371/journal.pone.0026442)
Supplement: Figure S2 — Mapping results of FFPE 2 sequences with Culture 2 and ten GenBank H. pylori strains. (PDF) [file pone.0026442.s002.pdf]

Supplementary Figure 2. Mapping results of FFPE 2 sequences with Culture 2 and ten GenBank *H. pylori* strains.

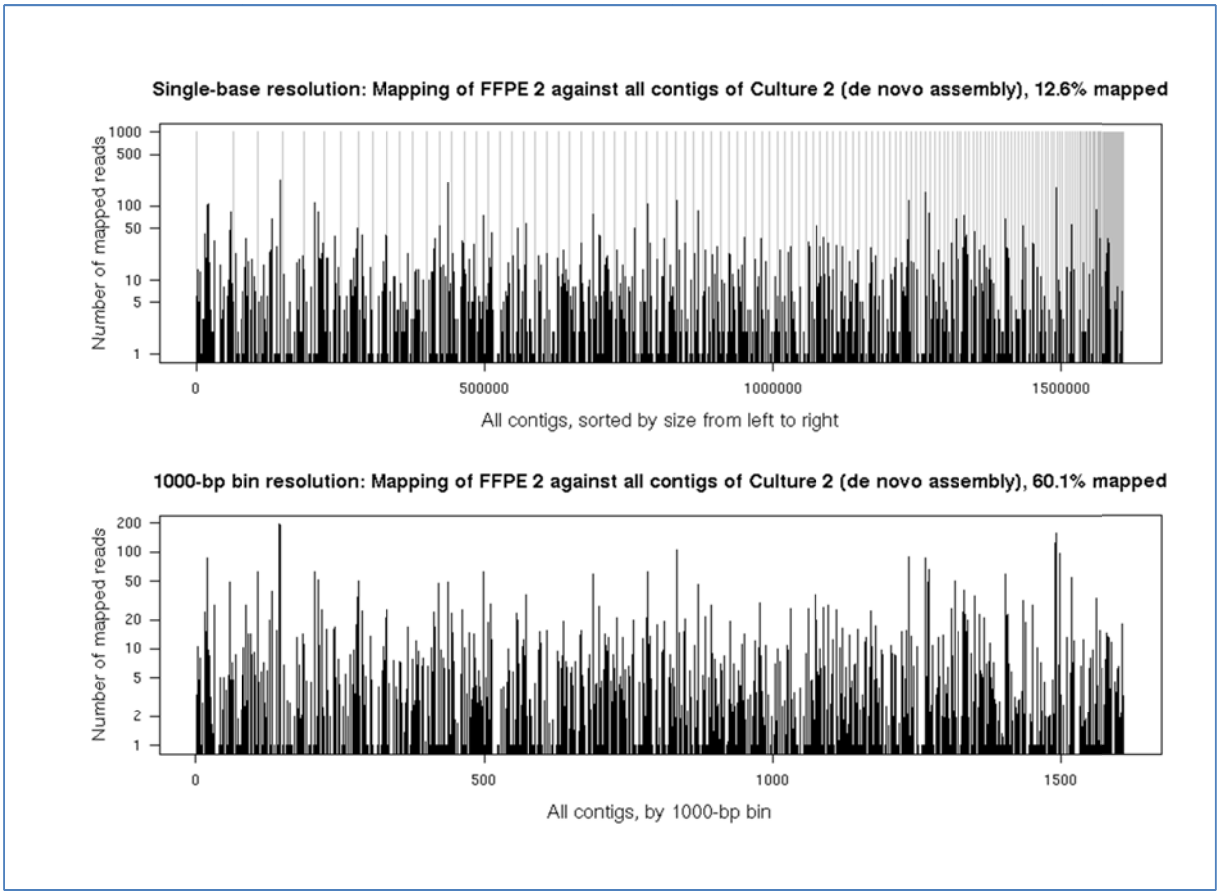

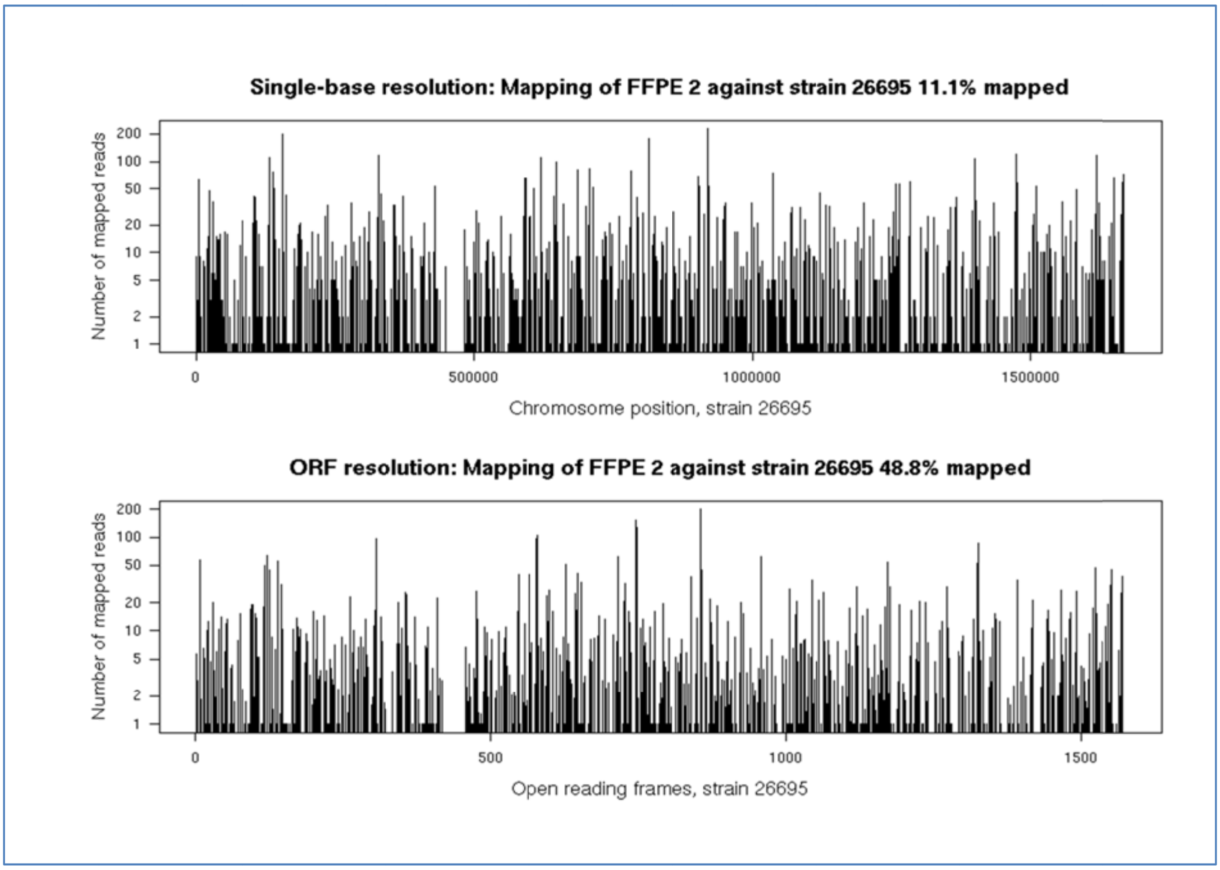

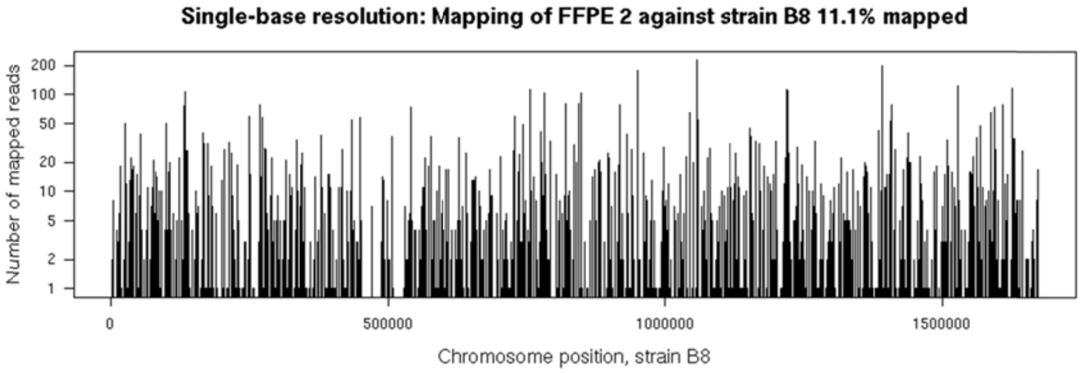
Single-base resolution: Mapping of FFPE 2 against strain B8 11.1% mapped
Number of mapped reads
200
100
50
20
10
5
2
1
0
500000
1000000
1500000
Chromosome position, strain B8

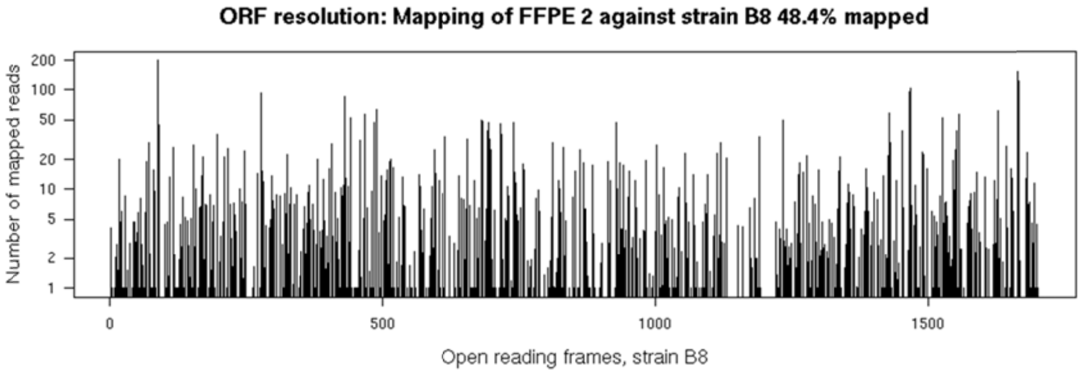
ORF resolution: Mapping of FFPE 2 against strain B8 48.4% mapped
Number of mapped reads
200
100
50
20
10
5
2
1
0
500
1000
1500
Open reading frames, strain B8

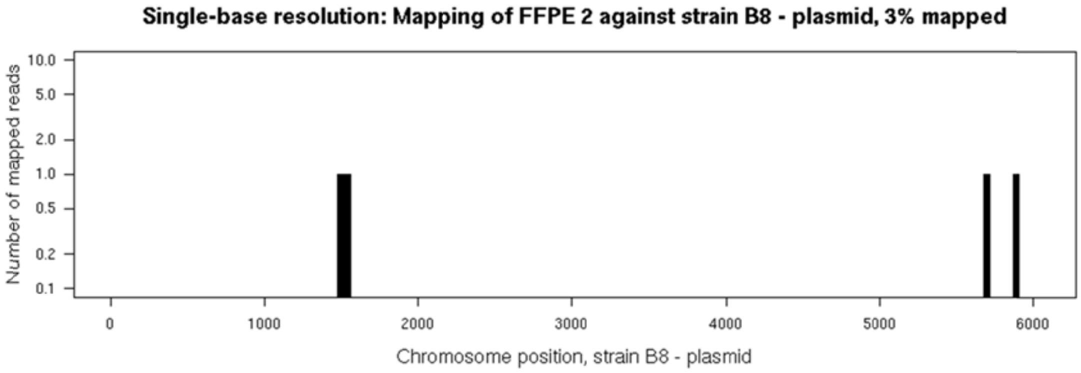
Single-base resolution: Mapping of FFPE 2 against strain B8 - plasmid, 3% mapped
Number of mapped reads
10.0
5.0
2.0
1.0
0.5
0.2
0.1
0
1000
2000
3000
4000
5000
6000
Chromosome position, strain B8 - plasmid

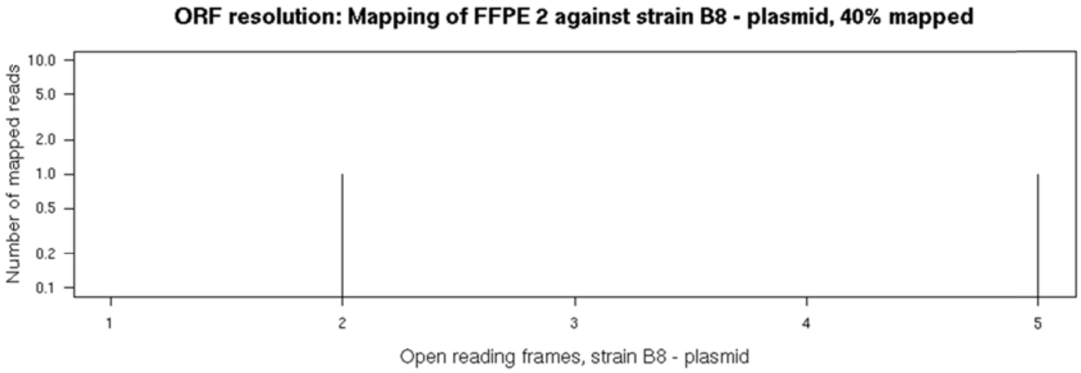
ORF resolution: Mapping of FFPE 2 against strain B8 - plasmid, 40% mapped
Number of mapped reads
10.0
5.0
2.0
1.0
0.5
0.2
0.1
1
2
3
4
5
Open reading frames, strain B8 - plasmid

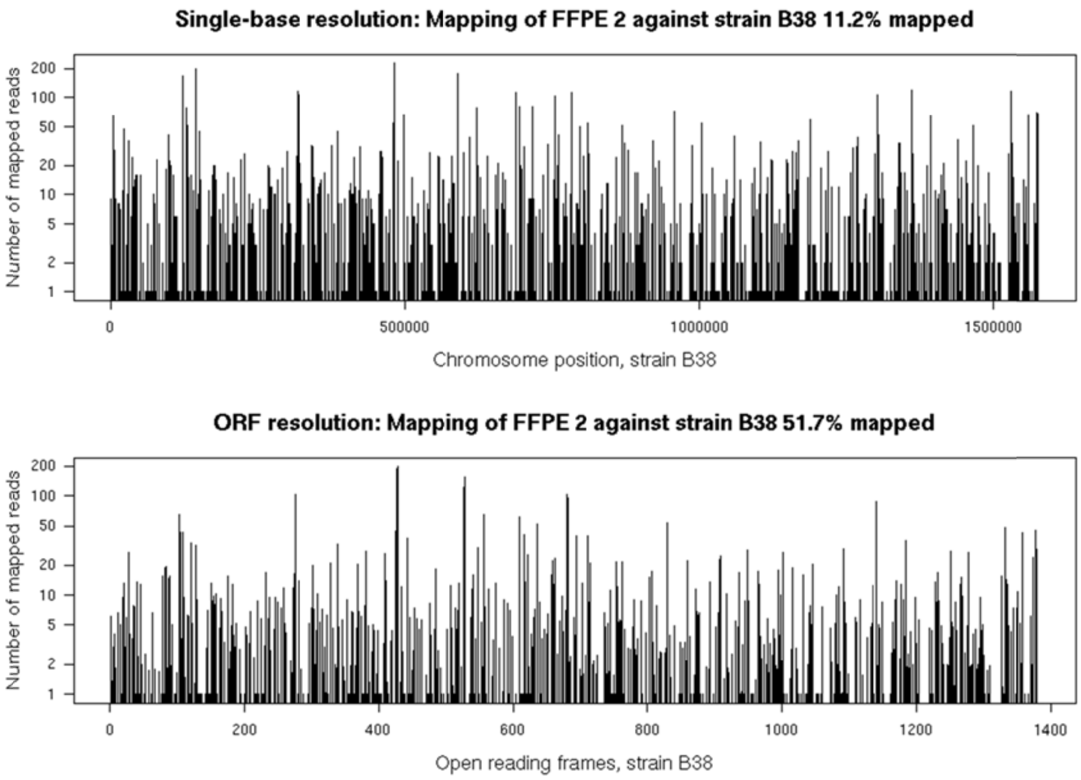
Single-base resolution: Mapping of FFPE 2 against strain B38 11.2% mapped
Number of mapped reads
1
2
5
10
20
50
100
200
0
500000
1000000
1500000
Chromosome position, strain B38
ORF resolution: Mapping of FFPE 2 against strain B38 51.7% mapped
Number of mapped reads
0
200
400
600
800
1000
1200
1400
Open reading frames, strain B38

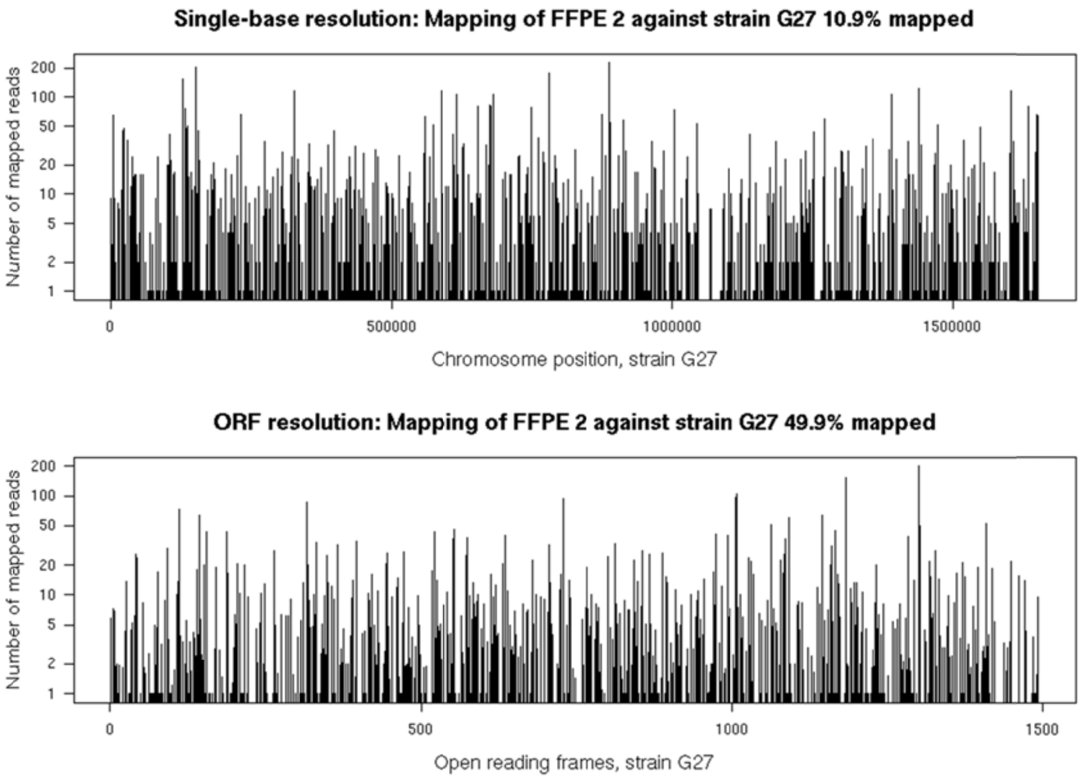
Single-base resolution: Mapping of FFPE 2 against strain G27 10.9% mapped
Number of mapped reads
1
2
5
10
20
50
100
200
0
500000
1000000
1500000
Chromosome position, strain G27
ORF resolution: Mapping of FFPE 2 against strain G27 49.9% mapped
Number of mapped reads
0
500
1000
1500
Open reading frames, strain G27

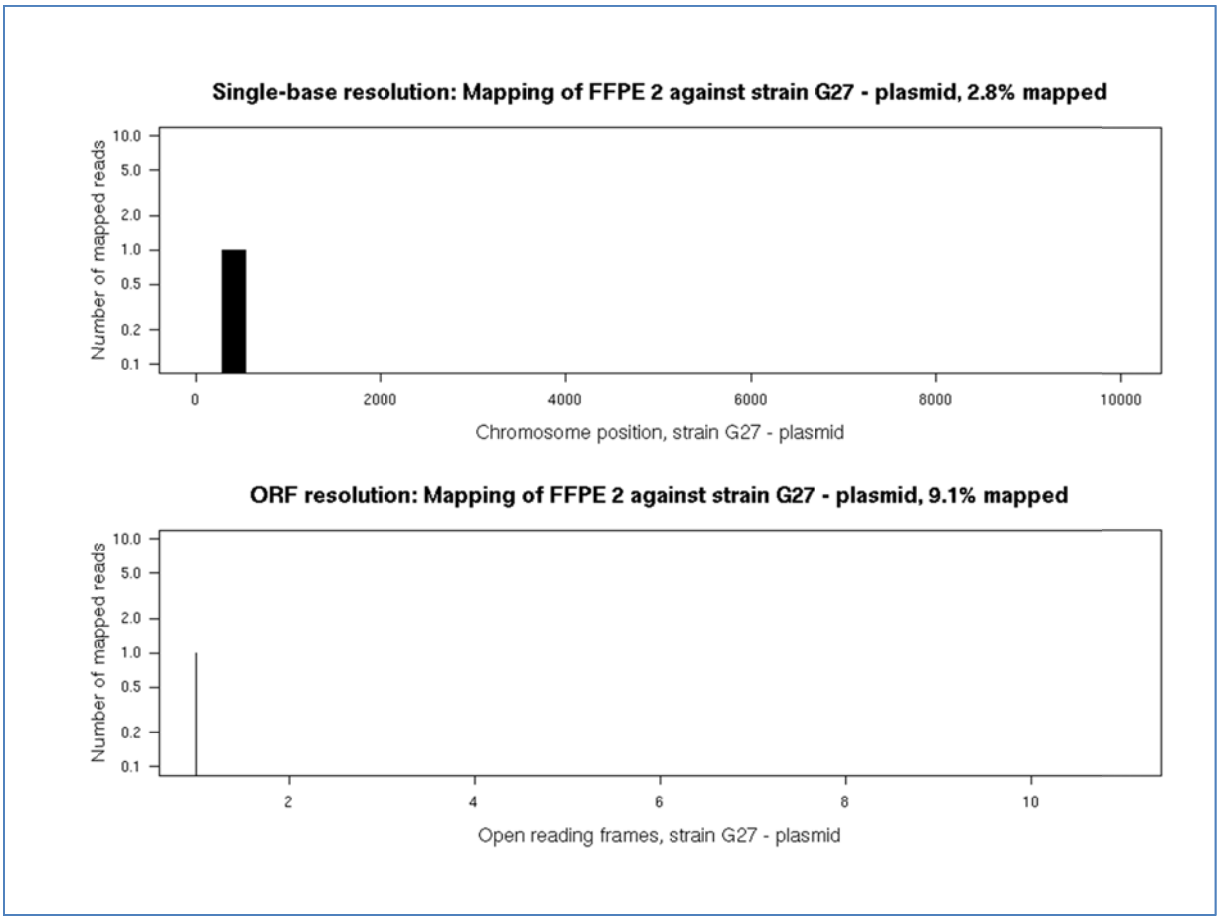
Single-base resolution: Mapping of FFPE 2 against strain G27 - plasmid, 2.8% mapped
Number of mapped reads
10.0
5.0
2.0
1.0
0.5
0.2
0.1
0
2000
4000
6000
8000
10000
Chromosome position, strain G27 - plasmid
ORF resolution: Mapping of FFPE 2 against strain G27 - plasmid, 9.1% mapped
Number of mapped reads
10.0
5.0
2.0
1.0
0.5
0.2
0.1
2
4
6
8
10
Open reading frames, strain G27 - plasmid

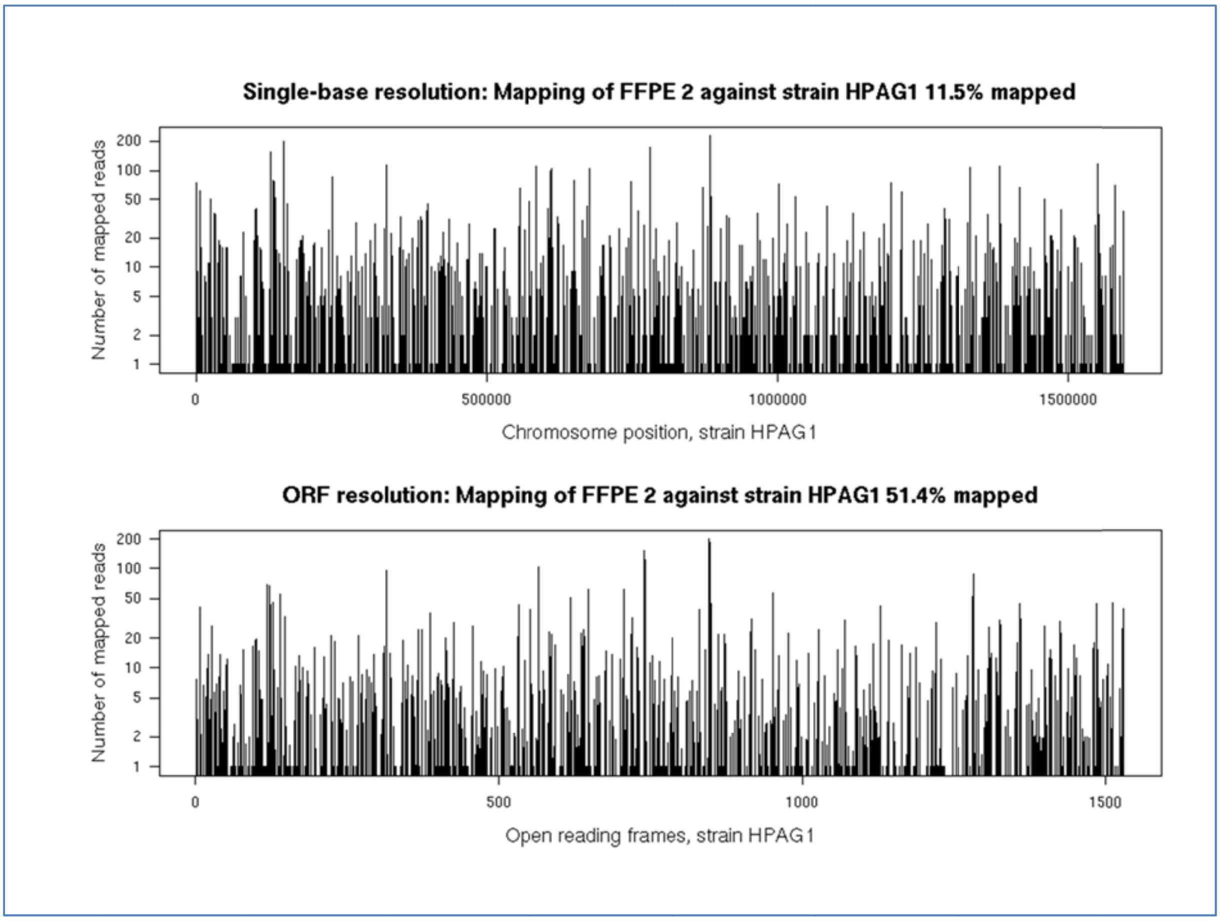
Single-base resolution: Mapping of FFPE 2 against strain HPAG1 11.5% mapped
Number of mapped reads
200
100
50
20
10
5
2
1
0
500000
1000000
1500000
Chromosome position, strain HPAG1
ORF resolution: Mapping of FFPE 2 against strain HPAG1 51.4% mapped
Number of mapped reads
200
100
50
20
10
5
2
1
0
500
1000
1500
Open reading frames, strain HPAG1

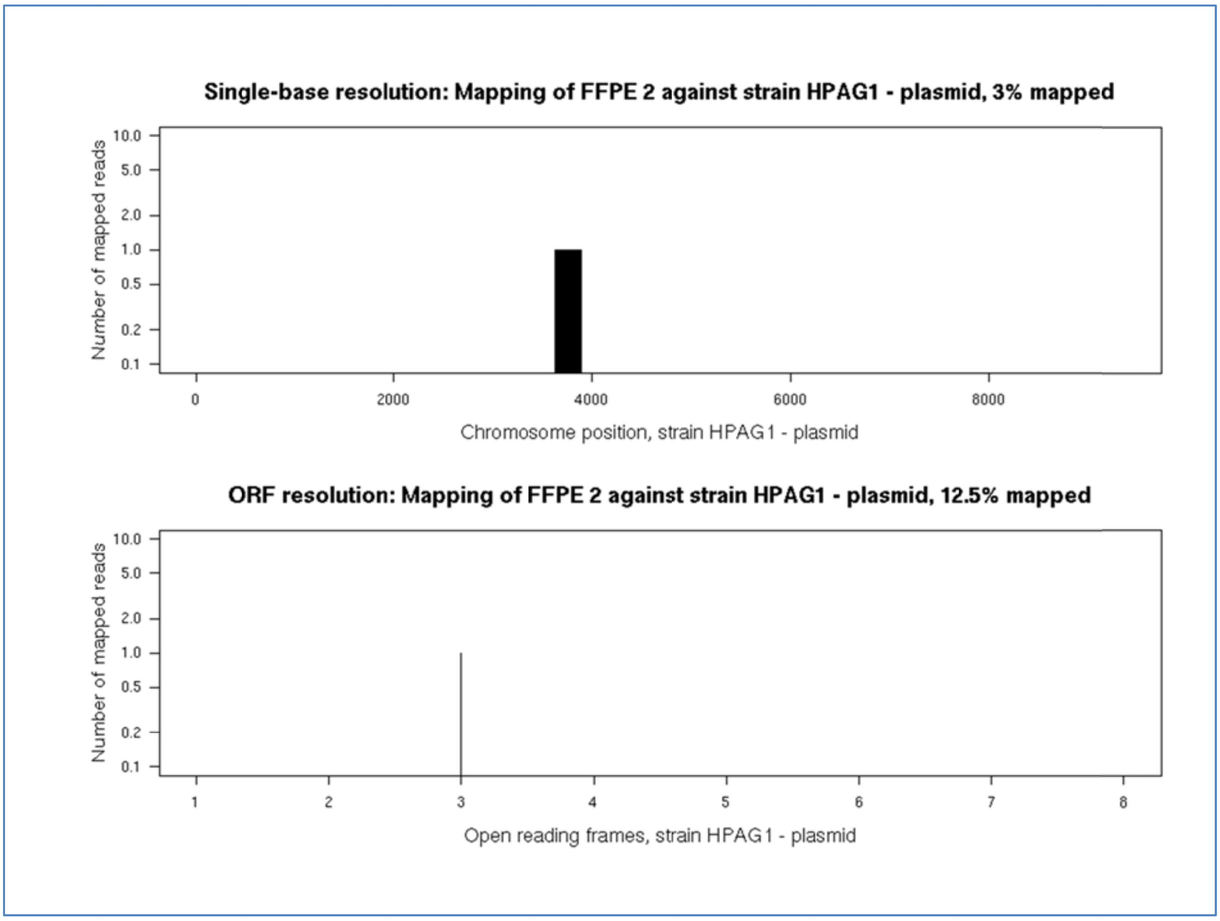
Single-base resolution: Mapping of FFPE 2 against strain HPAG1 - plasmid, 3% mapped
Number of mapped reads
Chromosome position, strain HPAG1 - plasmid
ORF resolution: Mapping of FFPE 2 against strain HPAG1 - plasmid, 12.5% mapped
Number of mapped reads
Open reading frames, strain HPAG1 - plasmid

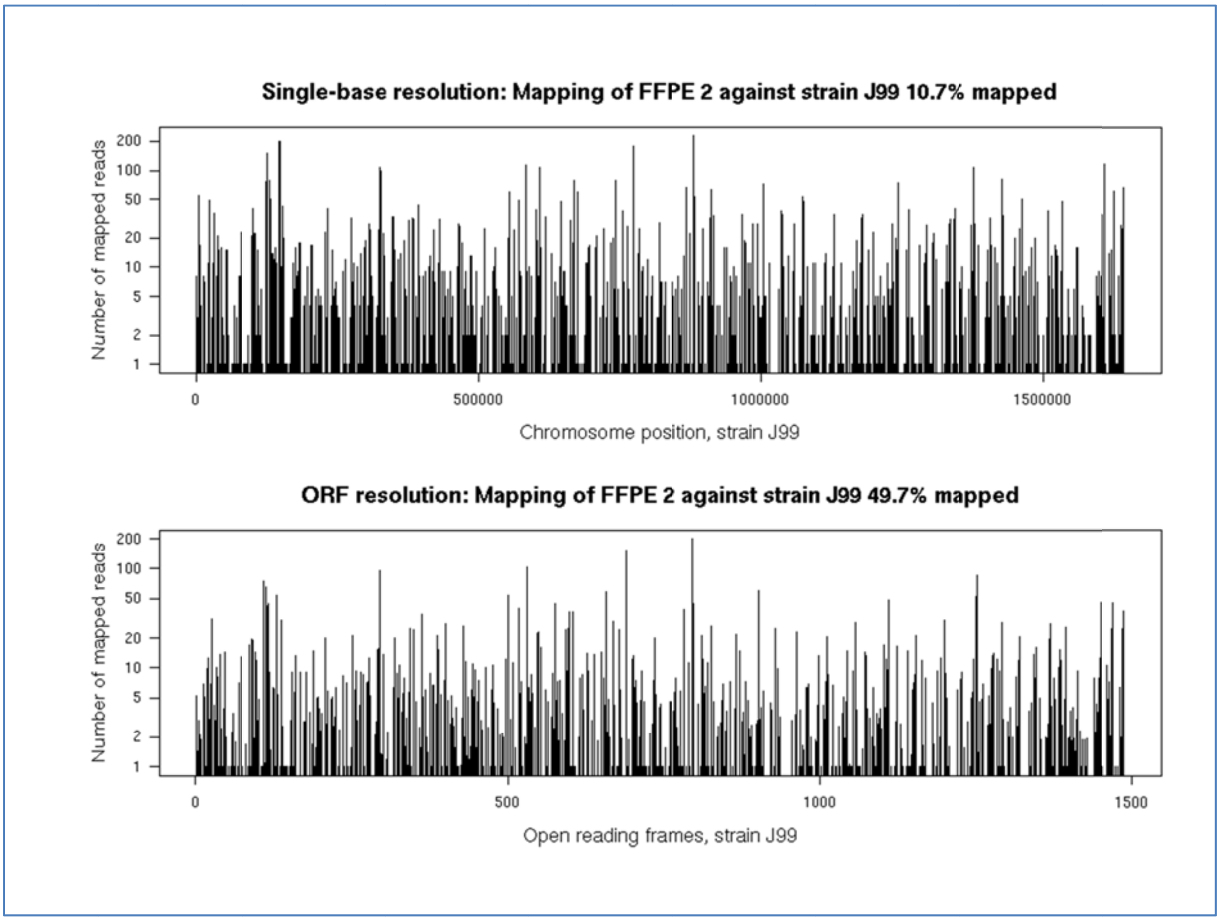
Single-base resolution: Mapping of FFPE 2 against strain J99 10.7% mapped
Number of mapped reads
Chromosome position, strain J99
ORF resolution: Mapping of FFPE 2 against strain J99 49.7% mapped
Number of mapped reads
Open reading frames, strain J99

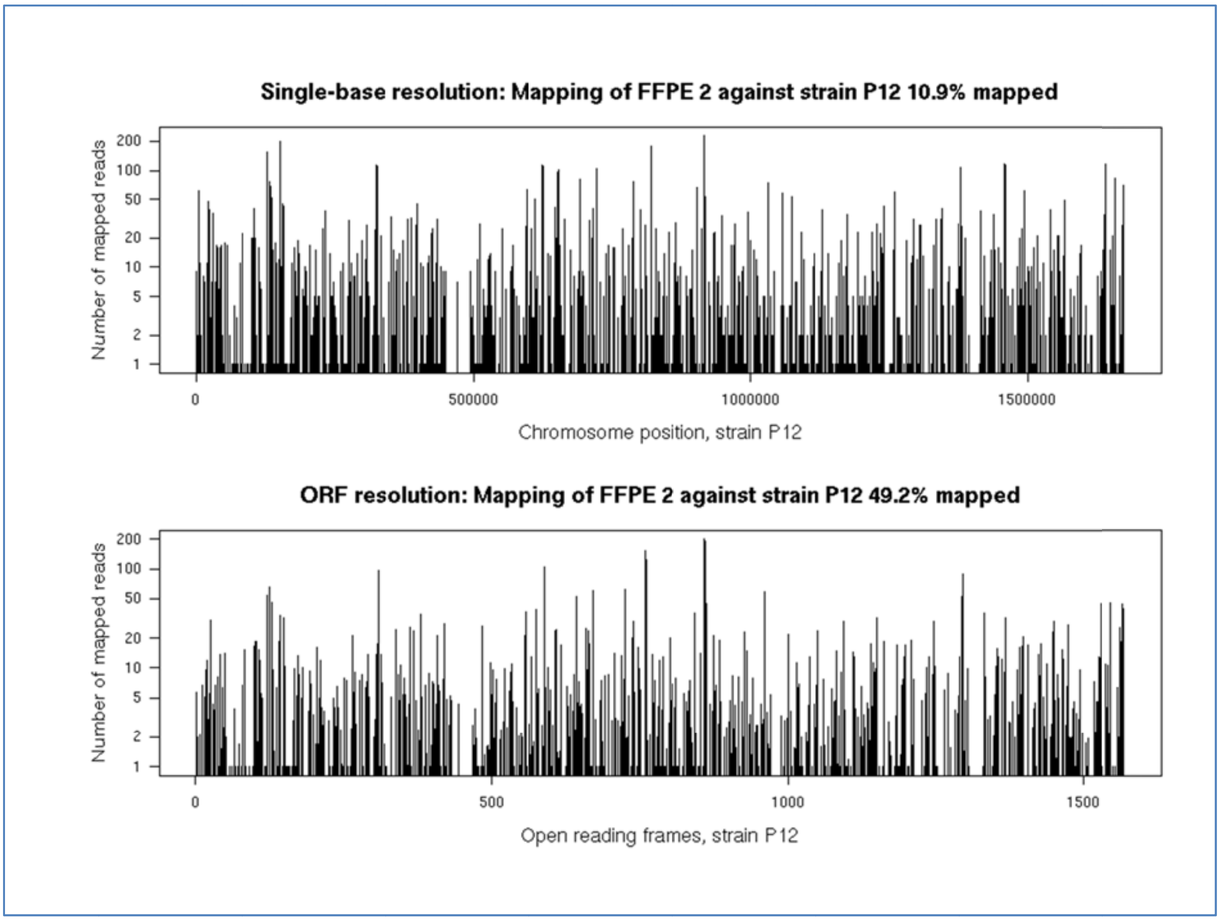

No read was mapped against P12 plasmid.

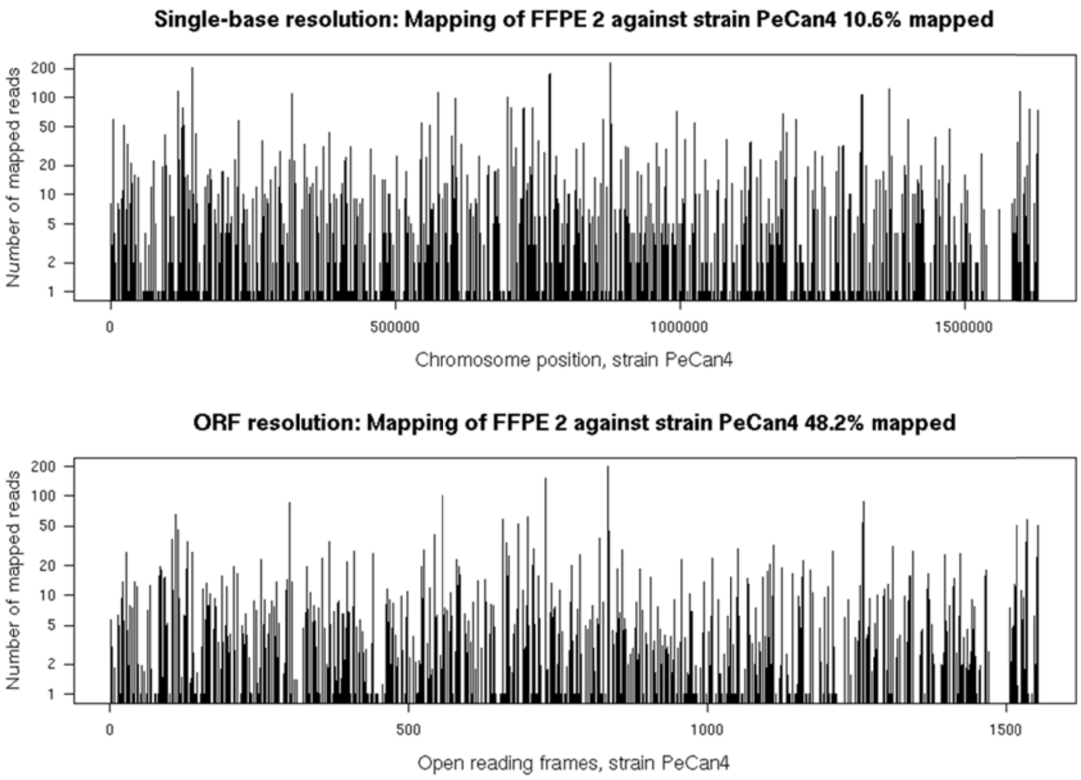

No read was mapped against PeCan4 plasmid.

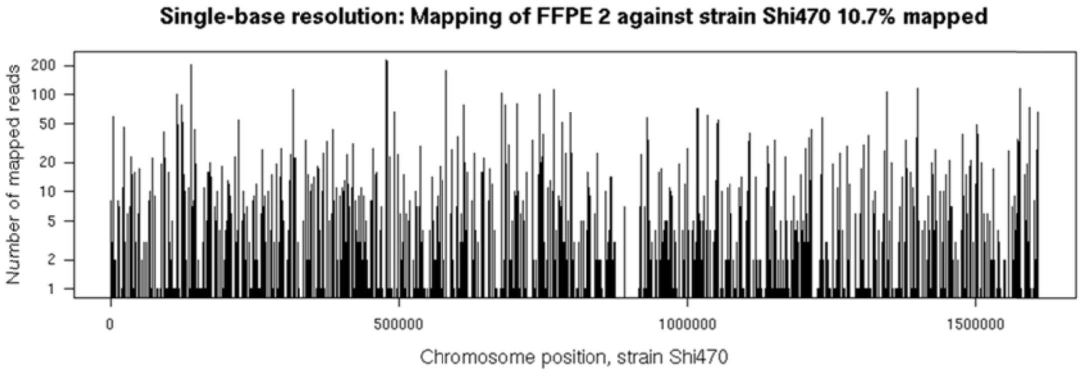
Single-base resolution: Mapping of FFPE 2 against strain Shi470 10.7% mapped
Number of mapped reads
1
2
5
10
20
50
100
200
0
500000
1000000
1500000
Chromosome position, strain Shi470

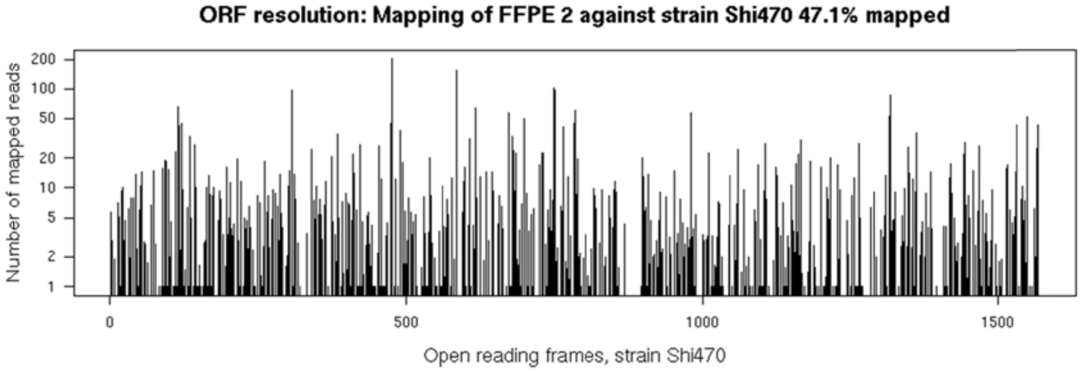
ORF resolution: Mapping of FFPE 2 against strain Shi470 47.1% mapped
Number of mapped reads
1
2
5
10
20
50
100
200
0
500
1000
1500
Open reading frames, strain Shi470

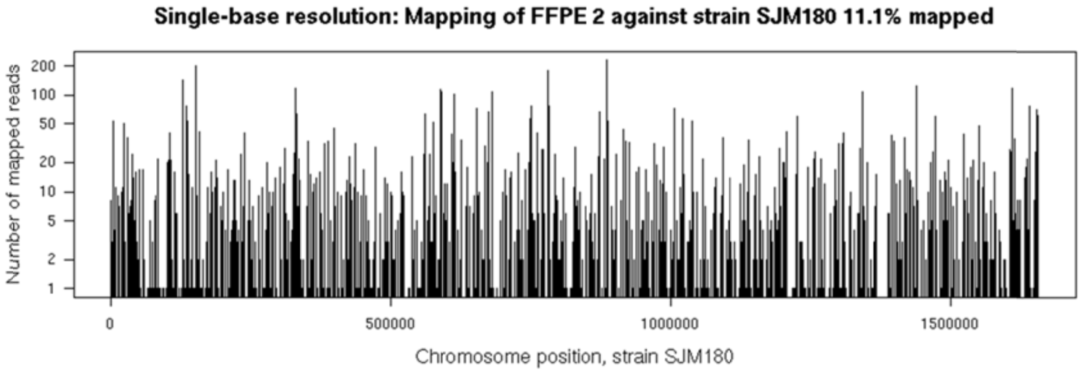
Single-base resolution: Mapping of FFPE 2 against strain SJM180 11.1% mapped
Number of mapped reads
1
2
5
10
20
50
100
200
0
500000
1000000
1500000
Chromosome position, strain SJM180

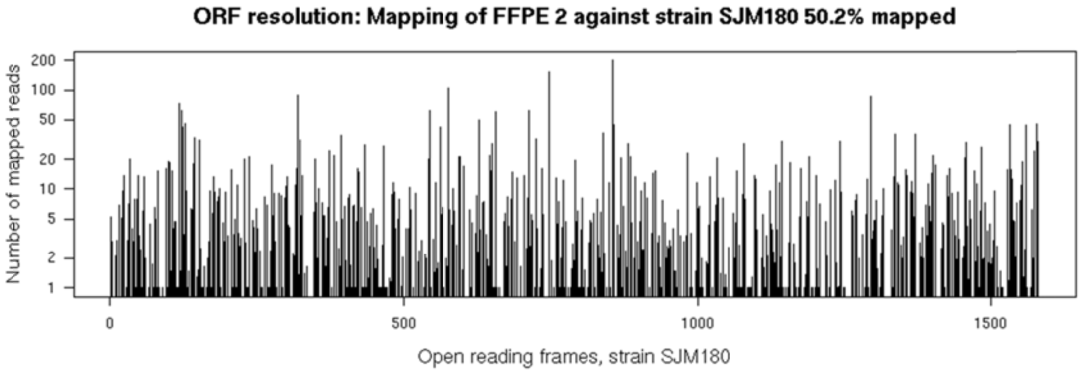
ORF resolution: Mapping of FFPE 2 against strain SJM180 50.2% mapped
Number of mapped reads
1
2
5
10
20
50
100
200
0
500
1000
1500
Open reading frames, strain SJM180
