# Supplementary material for: A Method for Metagenomics of Helicobacter pylori from Archived Formalin-Fixed Gastric Biopsies Permitting Longitudinal Studies of Carcinogenic Risk
Source: PLoS One. 2011 Oct 21;6(10):e26442. doi: 10.1371/journal.pone.0026442 (PMC3198776; doi:10.1371/journal.pone.0026442)
Supplement: File S1 — Blastx results of those FFPE 1 sequences aligned only with Culture 1 sequences. (DOC) [file pone.0026442.s003.doc]

Blastx results of those FFPE 1 sequences aligned only with culture 1 sequences:

hypothetical protein HPSA_08249

MobA-like protein

D-3-phosphoglycerate dehydrogenase

MobA-like protein

type II restriction enzyme

hypothetical protein pHel4_10

cytosine specific DNA methyltransferase

cytosine specific DNA methyltransferase

hypothetical protein pHel4_14

cytosine specific DNA methyltransferase

MobA-like protein

Type I restriction enzyme M protein

hypothetical protein HPLT_06630

hypothetical protein pHel4_11

IS606 transposase

IS606 transposase

iron-regulated outer membrane protein

hypothetical protein HPLT_00150

Hypothetical protein HPV225_0393

phosphatidate cytidylyltransferase

hypothetical protein HPSJM_00965

cytosine specific DNA methyltransferase

hypothetical protein HPF57_0280

IS606 transposase

IS606 transposase

AF326621_1 HP790-like protein

type III restriction-modification system methylation subunit

hypothetical protein pHel4_11

IS606 transposase

IS606 transposase

MobA-like protein

hypothetical protein HPLT_04360

IS606 transposase

hypothetical protein pAL202_04

hypothetical protein pHel4_11

hypothetical protein pHel4_11

hypothetical protein pAL202_04

hypothetical protein pHel4_11

hypothetical protein pHel4_11

MobA-like protein

hypothetical protein HPSA_08249

iron-regulated outer membrane protein

hypothetical protein pHel4_11

hypothetical protein HP0726

hypothetical protein HPB8_343

AF326614_1 JHP318-like protein

hypothetical protein HPF57_1402

IS606 transposase

hypothetical protein pHel4_11

hypothetical protein pHel4_11

glycerol-3-phosphate dehydrogenase

replication initiation protein A

IS606 transposase

IS606 transposase

hypothetical protein HP0726

hypothetical protein pAL202_04

hypothetical protein HpylHP_00362

hypothetical protein HPF30_0433

hypothetical protein HPGAM_00900

hypothetical protein pAL202_04

MobA-like protein

hypothetical protein HPSA_08249

Hypothetical protein HPV225_0378

IS606 transposase

IS606 transposase

hypothetical protein pHel4_11

hypothetical protein HPG27_724

IS606 transposase

hypothetical protein pHel4_11

hypothetical protein HPGAM_00900

MobA-like protein

hypothetical protein HPF57_0603

hypothetical protein pAL202_04

hypothetical protein HPLT_08389

hypothetical protein HpylHP_02056

hypothetical protein pHel4_11

hypothetical protein HpylH_16935

hypothetical protein HPLT_08389

dihydroorotase

MobA-like protein

hypothetical protein HPSJM_06760

hypothetical protein HPP12_0055

hypothetical protein pAL202_04

hypothetical protein pHel4_11

hypothetical protein HPSJM_02730

hypothetical protein pAL202_04

magnesium and cobalt transport protein

AF326614_1 JHP318-like protein

type III DNA modification enzyme (methyltransferase)

putative vacuolating cytotoxin VacA

hypothetical protein pHel4_11

type III restriction enzyme R protein (res)

hypothetical protein pAL202_04

hypothetical protein pHel4_11

hypothetical protein pAL202_04

hypothetical protein pHel4_11

hypothetical protein pHel4_11

hypothetical protein pAL202_04

hypothetical protein pHel4_11

hypothetical protein pAL202_04

hypothetical protein pAL202_04

MobA-like protein

hypothetical protein HPLT_08020

branched-chain amino acid transporter AzlD

hypothetical protein pHel4_11

hypothetical protein pHel4_11

hypothetical protein pHel4_12

hypothetical protein pAL202_04

MobA-like protein

hypothetical protein HPF57_0280

hypothetical protein pAL202_04

MobA-like protein

hypothetical protein pAL202_04

hypothetical protein pAL202_04

AF326623_1 JHP726-like protein

hypothetical protein pHel4_11

MobA-like protein

hypothetical protein HPF16_1332

hypothetical protein pHel4_10

replication initiation protein A

hypothetical protein HPKB_0065

hypothetical protein pHel4_11

hypothetical protein pHel4_11

replication initiation protein A

iron-regulated outer membrane protein

hypothetical protein pHel4_10

hypothetical protein pAL202_04

hypothetical protein pHel4_11

hypothetical protein

hypothetical protein pHel4_11

hypothetical protein pHel4_11

hypothetical protein pAL202_04

hypothetical protein pAL202_04

hypothetical protein pHel4_11

hypothetical protein pHel4_11

hypothetical protein pHel4_11

hypothetical protein HPF57_0603

hypothetical protein HpylHP_12210

transposase TnpB

hypothetical protein pHel4_11

MobA-like protein

hypothetical protein pHel4_11

hypothetical protein HPF32_0710

hypothetical protein pHel4_11

hypothetical protein pHel4_11

magnesium and cobalt transport protein

hypothetical protein pHel4_11

hypothetical protein HPAG1_1083

peptidyl-tRNA hydrolase

hypothetical protein HPF16_0063

hypothetical protein pHel4_11

MobD-like protein

MobD-like protein

hypothetical protein HPSA_00880

hypothetical protein hp908_1212

hypothetical protein pHel4_11

hypothetical protein pHel4_11

IS606 transposase

hypothetical protein HPLT_08304

hypothetical protein pHel4_11

hypothetical protein pHel4_11

hypothetical protein HPP12_0774

IS606 transposase

hypothetical protein pHel4_11

hypothetical protein pHel4_11

hypothetical protein HPF57_0280

hypothetical protein pHel4_11

magnesium and cobalt transport protein

hypothetical protein pHel4_11

hypothetical protein pHel4_11

hypothetical protein pAL202_04

IS606 transposase

hypothetical protein HELPY_0996

hypothetical protein pHel4_11

carbamoyl phosphate synthase small subunit

hypothetical protein pHel4_11

MobA-like protein

hypothetical protein pAL202_04

hypothetical protein pHel4_11

hypothetical protein pHel4_11

putative type III restriction enzyme R protein (Res)

hypothetical protein pHel4_11

replication initiation protein A

hypothetical protein

MobA-like protein

hypothetical protein pHel4_11

hypothetical protein pHel4_11

hypothetical protein pAL202_04

MobC

MobA-like protein

putative vacuolating cytotoxin(VacA)-like protein

hypothetical protein pHel4_11

hypothetical protein pAL202_04

hypothetical protein HPPC_01025

hypothetical protein pHel4_11

hypothetical protein pHel4_11

hypothetical protein pAL202_04

H64545 lipopolysaccharide 1,2-glucosyltransferase - Helicobacter pylori

MobA-like protein

hypothetical protein pHel4_11

MobA-like protein

pantoate--beta-alanine ligase

hypothetical protein pHel4_11

hypothetical protein pHel4_11

hypothetical protein pAL202_04

MobA-like protein

H64545 lipopolysaccharide 1,2-glucosyltransferase - Helicobacter pylori

transposase TnpB

hypothetical protein pHel4_11

magnesium and cobalt transport protein

hypothetical protein pHel4_11

replication initiation protein A

hypothetical protein pHel4_11

hypothetical protein pHel4_11

hypothetical protein pHel4_11

MobA-like protein

type III restriction enzyme

type I restriction enzyme R protein

hypothetical protein pAL202_04

hypothetical protein HPAG1_1333

hypothetical protein HPLT_05080

hypothetical protein pAL202_04

hypothetical protein pHel4_11

magnesium and cobalt transport protein

adenine-specific DNA-methyltransferase

hypothetical protein pHel4_11

hypothetical protein pAL202_04

hypothetical protein pAL202_04

H64545 lipopolysaccharide 1,2-glucosyltransferase - Helicobacter pylori

hypothetical protein HPF16_0063

hypothetical protein HPF16_0063

hypothetical protein pHel4_11

hypothetical protein pAL202_04

replication initiation protein A

primosome assembly protein PriA

replication initiation protein A

hypothetical protein pAL202_04

hypothetical protein HPSAT_04100

hypothetical protein pAL202_04

ubiquinol-cytochrome c reductase cytochrome b subunit

hypothetical protein HP0852

peptidyl-tRNA hydrolase

hypothetical protein pHel4_11

H64545 lipopolysaccharide 1,2-glucosyltransferase - Helicobacter pylori

hypothetical protein pHel4_11

phosphatidylserine decarboxylase

H64545 lipopolysaccharide 1,2-glucosyltransferase - Helicobacter pylori

hypothetical protein pAL202_04

hypothetical protein pAL202_04

hypothetical protein pAL202_04

hypothetical protein pHel4_11

hypothetical protein HPSA_08249

hypothetical protein pHel4_11

replication initiation protein A

H64545 lipopolysaccharide 1,2-glucosyltransferase - Helicobacter pylori

hypothetical protein HPB8_2

hypothetical protein pHel4_11

H64545 lipopolysaccharide 1,2-glucosyltransferase - Helicobacter pylori

IS606 transposase

hypothetical protein pHel4_11

hypothetical protein HPF32_0270

hypothetical protein pHel4_11

MobA-like protein

H64545 lipopolysaccharide 1,2-glucosyltransferase - Helicobacter pylori

hypothetical protein HPPC_01025

H64545 lipopolysaccharide 1,2-glucosyltransferase - Helicobacter pylori

IS606 transposase

hypothetical protein pHel4_11

hypothetical protein pAL202_04

hypothetical protein pAL202_04

hypothetical protein pHel4_11

hypothetical protein pHel4_11

hypothetical protein pHel4_11

hypothetical protein HPLT_08020

MobA-like protein

hypothetical protein pHel4_11

H64545 lipopolysaccharide 1,2-glucosyltransferase - Helicobacter pylori

hypothetical protein HPPC_01025

hypothetical protein pHel4_11

hypothetical protein HP0965

hypothetical protein HPF32_0280

hypothetical protein HPB128_165g1

hypothetical protein HpylH_06619

H64545 lipopolysaccharide 1,2-glucosyltransferase - Helicobacter pylori

hypothetical protein pHel4_10

hypothetical protein pHel4_11

MobD-like protein

enoyl-(acyl carrier protein) reductase

hypothetical protein pHel4_11

hypothetical protein pHel4_11

hypothetical protein HPSA_08389

replication initiation protein A

hypothetical protein pHel4_11

hypothetical protein HPP12_0982

3-methyladenine DNA glycosylase

hypothetical protein HPG27_941

hypothetical protein HPSAT_07909

hypothetical protein pHel4_11

hypothetical protein pHel4_11

hypothetical protein pHel4_11

hypothetical protein pHel4_11

Hypothetical protein HPV225_1071

hypothetical protein pAL202_04

protease DO
