# Supplementary material for: A Method for Metagenomics of Helicobacter pylori from Archived Formalin-Fixed Gastric Biopsies Permitting Longitudinal Studies of Carcinogenic Risk
Source: PLoS One. 2011 Oct 21;6(10):e26442. doi: 10.1371/journal.pone.0026442 (PMC3198776; doi:10.1371/journal.pone.0026442)
Supplement: File S2 — Blastx results of those FFPE 2 sequences aligned only with Culture 2 sequences. (DOC) [file pone.0026442.s004.doc]

Blastx results of those FFPE 2 sequences aligned only with culture 2 sequences:

membrane fusion protein of the hefABC efflux system HefB

hypothetical protein HPLT_06205

coproporphyrinogen III oxidase

transcriptional regulator

hypothetical protein HPLT_06205

hypothetical protein HPGAM_00180

hypothetical protein HpylH_06414

hypothetical protein HPGAM_00225

glutamate:Na+ symporter

hypothetical protein HPF32_1008

hypothetical protein HPLT_06205

hypothetical protein HPGAM_00180

IS606 transposase

hypothetical protein HPF57_0170

coproporphyrinogen III oxidase

cytosine specific DNA methyltransferase

ferrodoxin-like protein

hypothetical protein HPF32_1011

hypothetical protein HELPY_0409

hypothetical protein HPGAM_00225

hypothetical protein HPF32_1009

outer membrane protein SabA

hypothetical protein HPF32_1008

hypothetical protein HPLT_06205

hydrogenase expression/formation protein HypD

hypothetical protein HPLT_06205

hypothetical protein hp908_0781

hypothetical protein HPGAM_00180

outer membrane protein

hypothetical protein HpylH_14260

hypothetical protein

hypothetical protein HPB8_1617

hypothetical protein HPSH_04715

hypothetical protein HPIN_03770

hypothetical protein HPIN_03755

gamma-glutamyltranspeptidase

putative regulatory protein

hypothetical protein HPIN_03770

transposase homolog B

hypothetical protein HPF32_1011

hypothetical protein HPIN_03755

hypothetical protein HPLT_06205

hypothetical protein HPGAM_00225

hypothetical protein HPF32_1008

hypothetical protein HPLT_07130

virulence associated protein D (vapD)

hypothetical protein HPF32_1011

hypothetical protein HPIN_03755

hypothetical protein HPGAM_08055

hypothetical protein HPF32_0348

hypothetical protein HPLT_08190

cell division protein FtsA

hypothetical protein HPIN_03770

hypothetical protein HPPC_00820

hypothetical protein HPIN_04460

hypothetical protein HPGAM_06405

Holliday junction DNA helicase RuvA

putative vacuolating cytotoxin(VacA)-like protein

hypothetical protein HPSH_07785

hypothetical protein HPIN_03730

hypothetical protein HPIN_03730

hypothetical protein HpylHP_14160

hypothetical protein HPIN_03755

hypothetical protein HELPY_0318

hypothetical protein HPPC_07665

type II restriction enzyme

hypothetical protein HPF32_1011

Penicillin-binding protein 3

gamma-glutamyltranspeptidase

hypothetical protein HPIN_03730

hypothetical protein HPIN_03730

3-dehydroquinate synthase

hypothetical protein HPP12_0958

hypothetical protein HPIN_03755

sodium/glutamate symport carrier protein/glutamate permease

hypothetical protein HPIN_03730

hypothetical protein HPIN_03730

hypothetical protein HPF32_1011

hypothetical protein HPIN_03755

hypothetical protein HPPC_04505

hypothetical protein HPIN_03730

hypothetical protein HPSAT_07515
